# Effect of meso-and micro-level quality improvement interventions to improve intrapartum care and birth outcome in Nepal- stepped wedge cluster randomized trial

**DOI:** 10.1016/j.xagr.2026.100697

**Published:** 2026-09-16

**Authors:** Rejina Gurung, Omkar Basnet, Prajwal Paudel, Lea Kreyenbaum, Pratiksha Bhattarai, Abhishek Gurung, Honey Malla, Avinash K Sunny, Srijana Sharma, Anna Axelin, Mats Målqvist, Luong Nguyen Thanh, Ashish Kc

**Affiliations:** 1 Research Division (Gurung, Basnet, Bhattarai, Gurung, Malla, Sunny, Sharma), Golden Community, Chakupat, Nepal; 2 Department of Women’s and Children’s Health (Gurung, Målqvist, Thanh, KC), Uppsala University, Uppsala, Sweden; 3 Paropakar Maternity and Women’s Hospital (Paudel), Kathmandu, Nepal; 4 School of Public Health and Community Medicine, Sahlgrenska Academy (Kreyenbaum, KC), University of Gothenburg, Gothenburg, Sweden; 5 Stockholm Resilience Center (Axelin), Stockholm University, Stockholm, Sweden; 6 Department of Nursing Science (Thanh), University of Turku, Turku, Finland

**Keywords:** quality improvement interventions, high quality intrapartum care, intrapartum outcome, stepped wedge randomized control trial, Nepal

## Abstract

**BACKGROUND:**

High-quality intrapartum care benefits from implementation of quality improvement (QI) interventions. However, scale-up of meso- and micro-level QI interventions to improve health facility performance have shown mixed results in Nepal.

**OBJECTIVE:**

This study aims to evaluate the effect of such interventions to intrapartum care and intrapartum mortality (intrapartum stillbirth and first day neonatal death) in public health facilities of Nepal.

**STUDY DESIGN:**

A stepped-wedge cluster randomized design was applied to implement the SUSTAIN package, comprising meso- and micro-level QI interventions in 8 hospitals across all provinces of Nepal over a 21-month period. Each hospital had a 2-month window to introduce QI interventions in their governance and clinical processes. Women with gestational age ≥22 weeks were included with research teams established in each hospital to monitor mortality and health workers’ performance. For each outcome, hospital-specific incidence rate ratios (IRRs) were estimated by comparing event rates in the intervention period with those in the control period.

**RESULTS:**

The study enrolled, 55,325 and 53,578 women were enrolled in the control and intervention periods, respectively. The SUSTAIN package resulted in a statistically non-significant reduction (16%) in intrapartum mortality at the hospital level (IRR 0.84 (95% CI: 0.66, 1.08)), while at the individual level there was also statistically non-significant lower odd (37%) of mortality (aOR: 0.63 (0.38, 1.04), *P*<.0001)). Improvement in intrapartum care were consistent in most of care indicators with adherence to hand-washing increased by 2.36 fold (IRR 2.36 (95% CI: 1.03, 5.39)), fetal heart-rate monitoring increased by 1.55 fold (IRR 1.55 (95% CI: 1.10, 2.17)), delayed cord clamping increased non-significantly by1.14 fold (IRR 1.14 (95% CI: 0.96, 1.35)), immediate skin-to-skin contact increased by 9.92 fold (IRR 9.92 (95% CI: 3.21, 30.64)), early breastfeeding increased by 4.09 fold (IRR 4.09 (95% CI: 2.07, 8.11)), and bag-and-mask within 1 minute increased by 3.30 fold (IRR 3.30 (95% CI: 2.02, 5.40).

**CONCLUSION:**

A synergistic and multi-faceted QI package implementation can improve intrapartum care with a statistically non-significant reduction in intrapartum mortality even during the COVID-19 pandemic.

**Trial:**

https://doi.org/10.1186/ISRCTN16741720

**AJOG Global Reports at a Glance**

**Why was this study conducted?**

Poor quality of intrapartum care is now a major determinant of intrapartum mortality (intrapartum stillbirth and first day neonatal mortality) in low-and middle-income countries. While evidence-based practices for intrapartum care exist, literature shows that micro-level interventions, including provider training, often result in only moderate improvements in performance.

**Key findings**

The study shows that quality improvement at meso- (system) and micro- (individual) levels for the implementation of intrapartum care improves healthcare workers’ care practices and non-significantly reduces intrapartum mortality even during COVID-19 pandemic.

**What does this add to what is known?**

Multi-level QI interventions (system and individual level) are effective and scalable in resource-constrained settings without requiring advanced technology but rather investing in the foundations of health systems.

## Introduction

Despite significant progress in maternal and newborn health (MNH) over the past 2 decades, preventable intrapartum-related mortality (intrapartum stillbirth and first-day mortality) remains a major public health challenge, particularly in low- and middle-income countries (LMICs)^1^ where 99% of these deaths occur.^2^^,^^3^ After the significant reductions achieved during the Millennium Development Goal era, the decline in stillbirth rates has lagged.^3^ Of the 5.8 million maternal and newborn deaths and stillbirths that occur each year.^2^^,^^3^ Half of these occur during the intrapartum period, and the majority are due to preventable causes.^3^^,^^4^ International frameworks such as the Sustainable Development Goals (SDGs) and Every Newborn Action Plan (ENAP) have set ambitious targets to reduce global neonatal mortality to 12 or fewer per 1000 births by 2030. Suboptimal quality of intrapartum care, including delayed recognition of fetal distress, inadequate monitoring during labor, and ineffective neonatal resuscitation, continues to reduce survival gains, even in settings where facility-based deliveries have increased.^5^^−^^7^ This requires a shift in focus from simply increasing access to care to ensuring quality services.^8^^,^^9^

A critical “know-do” gap exists in MNH. While cost-effective, evidence-based practices are well-documented, translating these standards into routine clinical practice remains a major challenge.^3^^,^^10^ Evidence from countries like India has shown that massively increasing facility-based deliveries do not automatically reduce mortality if the quality of care is poor.^9^ In many LMICs, mothers and children receive less than half of recommended clinical actions during a typical visit, and 60% of preventable deaths are now attributed to poor-quality care rather than a lack of access.^6^^,^^11^

Nepal has made substantial gains in maternal and neonatal survival, with institutional births increasing four-fold between 2001 and 2016.^12^ However, neonatal deaths and stillbirths still occur frequently due to poor-quality of intrapartum care.^13^ There is an urgent need for systematic solutions that can stabilize and improve care quality even in resource-constrained or crisis settings.^8^

Quality improvement (QI) is defined as achieving better outcomes through changing provider behavior and organizational culture using systematic methods.^3^^,^^6^ Recent research suggests that mutually reinforcing packages of interventions rather than individual or parallel strategies are most effective at reducing prematurity-related mortality.^14^ Effective QI requires action across multiple levels: the micro-level (health worker skills and motivation), the meso-level (hospital leadership and governance), and the macro-level (national policy and financing).^8^ While many studies have focused on micro-level performance, there is a lack of evidence specifically linking scaled-up QI packages to improved intrapartum outcomes.^15^, ^16^, ^17^

This study primarily aims to address these knowledge gaps by determining whether the SUSTAIN package, a micro- and meso-level QI intervention, can effectively reduce intrapartum-related mortality (intrapartum stillbirth and first day neonatal mortality), and secondly improve the quality of intrapartum care along with vital process indicators across selected public hospitals in Nepal.

## Methods

*Study Design:* This is a stepped-wedge, cluster-randomized controlled trial (SW-CRT) in 8 public hospitals across Nepal, and ensuring geographic representation.^18^ While 2 out of the 8 hospitals were from the same province, the others were from different regions. Hospitals were considered eligible if they reported ≥3000 deliveries annually and had functional neonatal and obstetric units. The intervention remained the same across all hospitals, but resource allocation varied depending on the delivery volume to account for logistical and human resource differences. Each hospital represented a cluster and was randomized in a cross-sectional wedge design, with an intervention transition period of 2 months per hospital with an exception for 1 hospital in which this period was 1 month (Figure 1).

**Figure 1:**
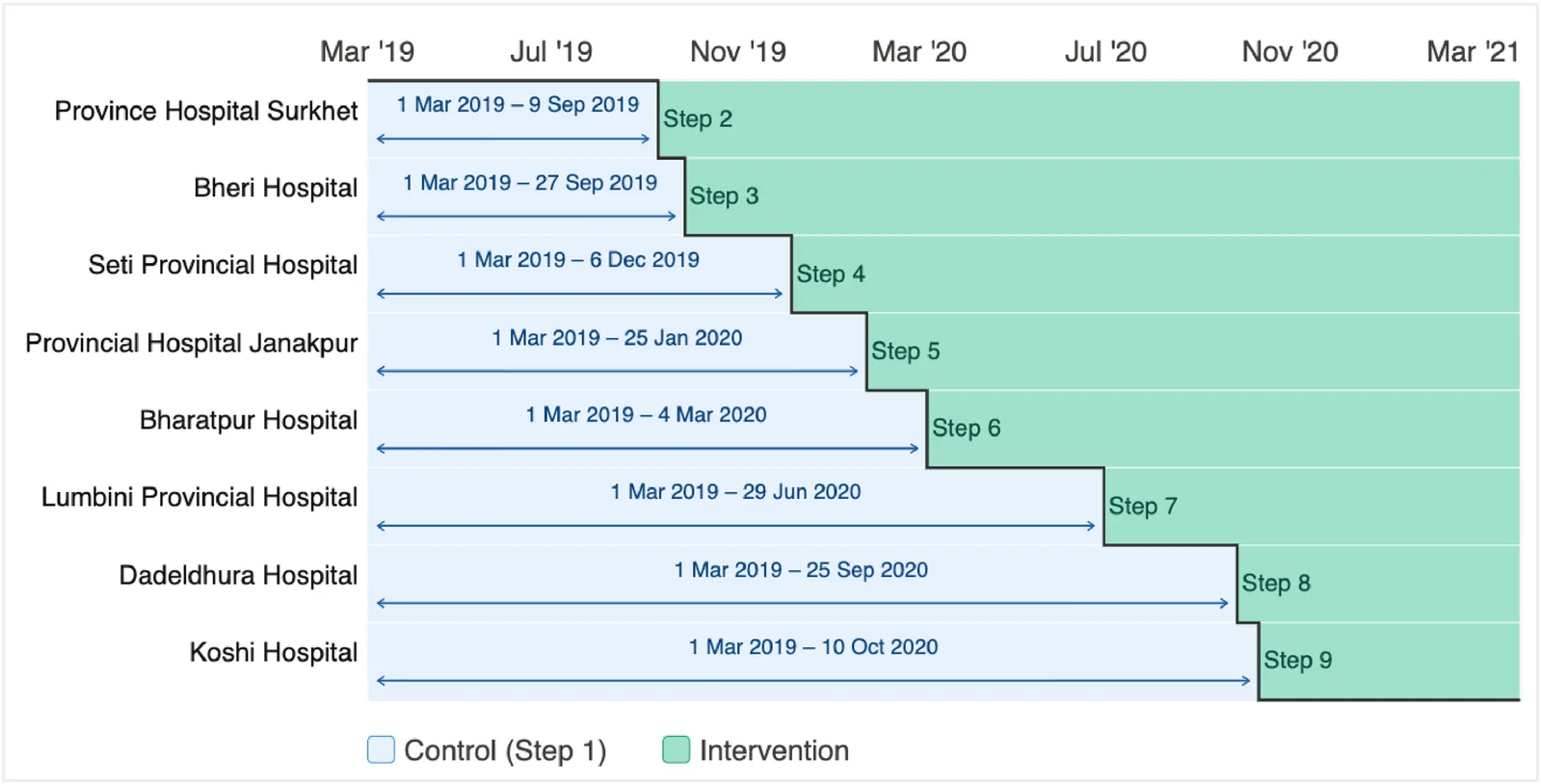
SUSTAIN implementation timeline: stepped wedge cluster randomized control trial *Gurung. Effect of meso-and micro-level quality improvement interventions to improve intrapartum care and birth outcome in Nepal- stepped wedge cluster randomized trial. Am J Obstet Gynecol 2026.*

Ethics approval was obtained from the Nepal Health Research Council (ref 2019/2867) with clinical trial registered ISRCTN [1674172], prospectively registered 02/03/2019.

*Participants:* For the study, the research team selected 8 public referral hospitals representing all provinces of the country. In each hospital, the study participants included women who came for delivery at the hospital aged ≥16 years, with a gestational age of ≥22 weeks who were admitted with detectable fetal heart sounds and gave consent to participate in the study. Exclusion criteria were the deliveries that occurred outside of the hospital, decline of consent, and gestational age <22 weeks.

*Randomization and Masking:* The Principal Investigator (PI) generated a random sequence in excel sheet for the 8 hospitals to determine the temporal stepped-wedge sequence of the clusters. Based on this sequence, the introduction of the intervention was decided. Blinding was not possible. Each hospital was informed about the enrolment of the intervention and its duration.

*Intervention:* The SUSTAIN QI package was designed to empower HCWs to improve intrapartum and immediate postpartum care. The intervention was delivered on 2 interconnected layers. At meso-level, the local intervention team signed a memorandum of understanding (MoU) the Ministry of Health and Population at the central level, followed by MoUs with each provincial ministry to secure administrative commitment. Next, we ran bottleneck-analysis workshops with medical superintendents, department chiefs of pediatrics, obstetrics and gynecology and nursing chiefs so that local priorities (poor quality of care and high perinatal mortality) were reflected in the subsequent work-plan. A readiness assessment evaluated existing staff numbers, equipment stocks and supplies, clinical guidelines and protocols, and clinical workflow in the maternity and newborn care units, and its results were presented to the hospital management including medical superintendent, Head of Department (HoD) and hospital development committee in separate group during a 1-day session that detailed the sequence of activities and defined accountability indicators.

At micro-level, every obstetric and neonatal care provider underwent 4 days of competency-based training in basic neonatal resuscitation and essential newborn care along with 1 dedicated day on quality improvement frameworks. A portable fetal heart monitors (Moyo®) and neonatal heart-rate meters (NeoBeat®) were introduced to identify fetal distress during labour and to improve neonatal resuscitation. The Safer Births Bundle, including upright bag-mask devices and NeoNatalie® live training manikins, was placed in each delivery room and weekly skill drills were scheduled. Thereby, every drill lasted 30 minutes and was immediately followed by debriefing using the Liveborn® application, which displayed compression rate, ventilation fraction and heart-rate response in real time. We also recommended 1 mentor in each hospital as a focal person for QI process based on their engagement and performance during the training. The mentor, either pediatrician or ward nursing in-charge was then decided by the medical superintendent or HOD in each hospital. A local QI team used Plan-Do-Study-Act (PDSA) cycles to review drill data every 2 weeks, set goals for the upcoming period, and document corrective actions. Project mentors visited each site twice a month to coach teams, verify that fidelity targets (≥80% of scheduled mentor visits and ≥75% of staff drill participation) were met, and upload aggregated indicators to a central dashboard. All the data collected daily on intrapartum and newborn care including skill drill were submitted the same day to the central data storage system. Once the data is submitted, the data access is restricted only to the data manager and the investigator. Daily updates of the data collection were done through an instant messaging applications (viber) group and weekly review meetings were conducted with data collectors. For any incomplete and inconsistent data entry, data manager verified the data during review meetings and handled it. Meeting minutes recorded any change processes. In addition, trainers and project team members did periodic on-site and remote monitoring which included documenting and addressing technological errors, quality data collection, skill drill training and PDSA meetings focused on intrapartum monitoring and neonatal resuscitation. An independent team from a Finnish University evaluated the implementation upon completion.^19^^,^^20^

Prior to data collection, all data collectors received comprehensive training on research methodology, principles and ethics. All of them received device training and were also trained in quality-of-care framework and the study proposal.

*Outcomes:* The trial had 1 primary outcome and 5 secondary outcomes, all assessed in the 8 participating hospitals.

*Primary Outcome:* Intrapartum-related mortality is defined as the incidence of intrapartum stillbirth and first day mortality per 1000 births among enrolled women. The metric is an incidence-rate ratio (IRR) (intervention vs. control) estimated over the entire trial period.

*Secondary Outcomes:* Six process indicators were included as secondary outcomes, hand-washing (hand-washing as per WHO recommended 6 steps), fetal heart-rate monitoring, delayed cord clamping, immediate skin-to-skin contact, early breastfeeding, and Bag- and Mask Ventilation (BMV) within 1 Minute (Table 1). All secondary outcomes are expressed as proportions aggregated at hospital level and analyzed as relative risks (RR), comparing pre-intervention with the post-intervention period. For Seti hospital, the intervention effect on all secondary outcomes (except fetal heart rate monitoring) could not be estimated, as there were no eligible pre-intervention denominators (see Figure 5).

**Table 1:** Definition of secondary outcomes and measurements

	Outcomes	Definition	Measurement
1	Hand-Washing	Hand-washing with soap before examining the mother and/or newborn as per WHO recommended 6 steps	Clinical Observation
2	Fetal Heart Rate Monitoring	Monitoring of fetal heart rate every 30 minutes before delivery	Patient Chart and Clinical Observation
3	Delayed Cord Clamping	Clamping of the umbilical cord of the newborn 60 seconds after the delivery	Clinical Observation
4	Immediate Skin to Skin Contact	Placing the newborn baby directly to the mother’s open chest or abdomen after birth	Clinical Observation
5	Early Breastfeeding	Early initiation of breastfeeding to the baby within 1 hour of birth	Clinical Observation
6	BMV within 1 Minute	BMV within 1 minute after birth among non-breathing newborns who were moved to the newborn corner for ventilation	Clinical Observation

Gurung. Effect of meso-and micro-level quality improvement interventions to improve intrapartum care and birth outcome in Nepal- stepped wedge cluster randomized trial. Am J Obstet Gynecol 2026.

*Statistical Analysis:* The intervention was employed in the same hospital, meaning every hospital was included in both the control and intervention groups. This design produced correlated data between the arms. For binary correlated data, the one-sided McNemar’s test was considered for sample size calculation. Considering a statistical power of 90%, a significance level of 5% one sided, and an average cluster size of 4800 deliveries, the sample size was calculated. To adjust for the cluster effect, the sample size was adjusted by the design effect, which was chosen as a proxy of 1.71 based on Nepal’s Demographic Health Survey (NDHS) 2016.^21^ This design effect was updated based on the average cluster size. The design effect for neonatal mortality rate (NMR) in NDHS 2016 was reported as 1.12 for an urban sample with an average cluster size of 799. Based on this information, the intra-cluster correlation coefficient (ICC) of 0.00015 was calculated, which gave a design effect of 1.71 for an average cluster size of 4800. A 7% loss to follow-up was also considered to reach the final sample size of 31,259 deliveries in each group. This number was proportionally allocated among the hospitals. The STATA command “power” was used for necessary calculations.

Descriptive analyses were first undertaken to summarize participant and facility characteristics. Outcomes were analyzed at hospital level by aggregating the number of events and the corresponding denominators separately for the control and intervention periods. For each outcome, hospital-specific incidence rate ratios (IRRs) with 95% CIs were estimated by comparing event rates in the intervention period with those in the control period. Pooled estimates were then obtained using random-effects meta-analysis. For secondary process outcomes, outcome-specific eligibility criteria were applied before aggregation, and pooled estimates were generated separately for each outcome. Missing or inapplicable observations for a given indicator were excluded from that specific analysis.

To examine factors associated with intrapartum mortality, a multivariable mixed-effects logistic regression model was fitted with intrapartum mortality as a main binary outcome. Covariates included intervention period, maternal age, ethnicity, maternal education, household wealth quintile, singleton versus twin pregnancy, admission complication, and preterm birth. Since, the trial was conducted during the COVID-19 pandemic, we also did the calendar adjustment. To account for clustering of participants within hospitals, hospital was included as a random intercept. Effect estimates are presented as adjusted odds ratios (aORs) with 95% CIs. The regression analysis used a complete-case approach. Participants with missing data for intrapartum mortality or for any covariate included in the model were excluded from this analysis. No imputation of missing data was undertaken. As this analysis was exploratory and aimed to identify predictors of intrapartum mortality, no non-inferiority margin was applicable. No interim analyses or formal stopping guidelines were specified for this model.

## Results

All 8 hospitals started in the control phase. The intervention was introduced sequentially according to the randomized stepped-wedge schedule, with hospitals transitioning one at a time until all clusters had implemented the intervention. The rollout occurred between September 2019 and March 2021 (Figure 2). During the control periods, 55,325 women were enrolled, and during the intervention periods, 53,578 women were enrolled.

**Figure 2:**
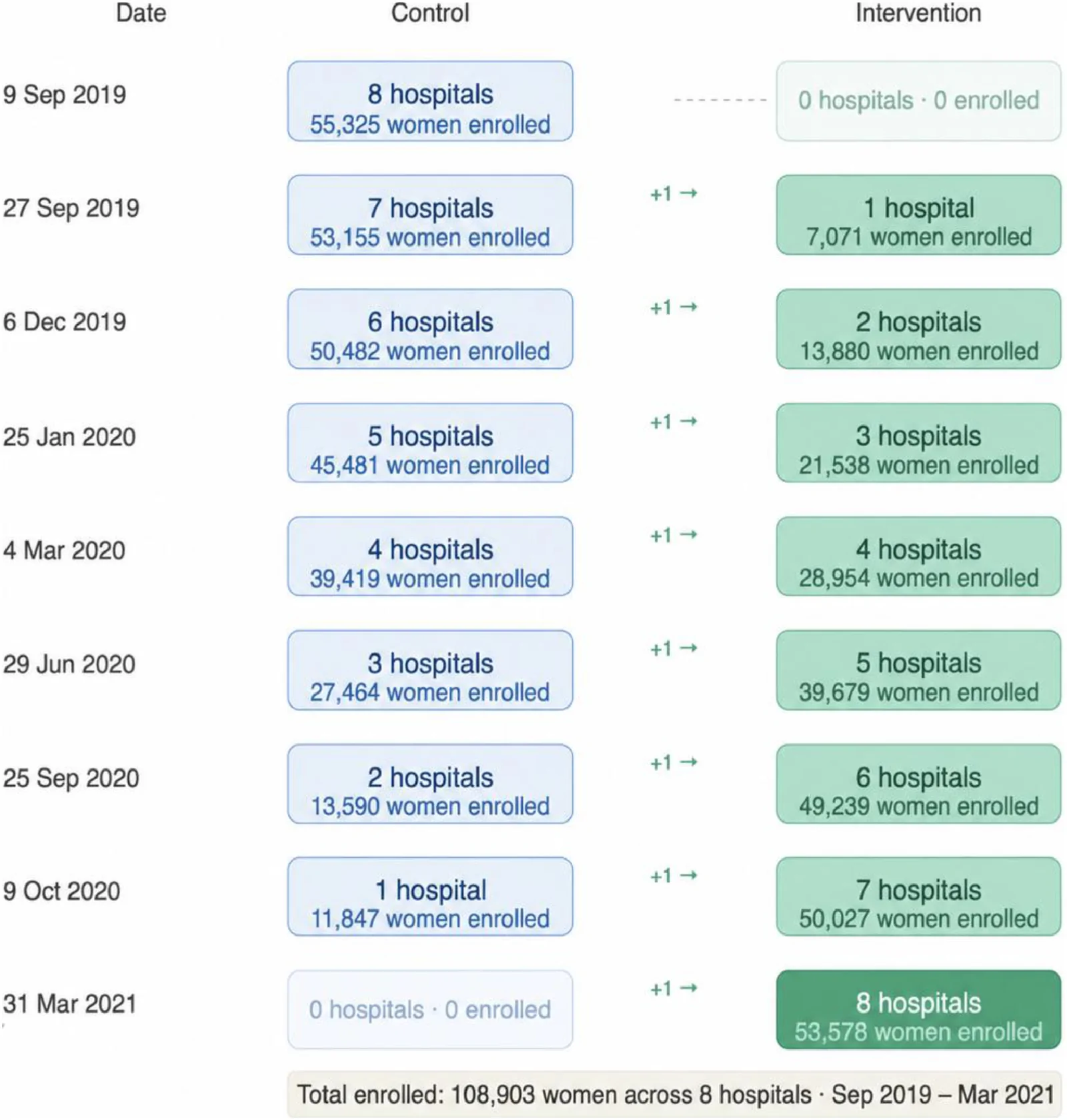
Consort flow chart diagram *Gurung. Effect of meso-and micro-level quality improvement interventions to improve intrapartum care and birth outcome in Nepal- stepped wedge cluster randomized trial. Am J Obstet Gynecol 2026.*

The mean maternal age was similar among both study period (24.0 ± 4.2 vs 24.3 ± 4.4). Women aged <20 years decreased from 5808 (10.7%) to 5191 (9.8%) and those aged 20 to 35 years increased from 47,869 (88.1%) to 47,030 (88.6%), similarly enrollment of women >35 years increased from 672 (1.2%) before intervention to 856 (1.6%) after intervention period. Regarding parity, Nulliparous women decreased from 24,017 (44.3%) to 19,900 (37.5%) and multiparous women increasing from 10,709 (19.8%) to 13,346 (25.2%) primiparous women were slightly increased 19,469 (35.9%) vs 19,784 (37.3%). Ethnic distribution was similar among both study periods, with advantageous groups at 18,205 (33.1%) vs 17,226 (33.4%) and disadvantaged groups at 36,784 (66.9%) vs 35,352 (66.6%). The mean gestational age was similar (38.5±1.8 vs 38.6±2.0 weeks), with preterm births slightly decreasing from 3594 (7.5%) to 3817 (7.2%). Mean birth weight increased (2889.0±507.6 vs 2948.5±518.1 g), with low birth weight declining from 6850 (12.7%) to 5534 (10.4%). Mean BMI decreased (24.10±6.4 vs 22.3±4.9) within the study period with a higher proportion in the normal range 1031 (92.7%) vs 2858 (95.6%). Wealth distribution showed a moderate shift, with the lowest quintile increasing 5948 (17.8%) vs. 7086 (22.5%) (Table 2).

**Table 2:** Characteristics of participants recruited in the study before and after intervention

Characteristics	Before intervention	After intervention
	n=55 325	n=53 578
Women’s age (years)*^,^^a^ **(n=107,426)**	**24·0±4·2**	**24·3±4·4**
<20 years	5808 (10·7%)	5191 (9·8%)
20−35 years	47,869 (88.1%)	47,030 (88.6%)
>35 years	672 (1.2%)	856 (1.6%)
Parity*^,^^b^ **(n=107,225)**		
Nulliparous	24 017 (44·3%)	19 900 (37·5%)
Primiparous	19 469 (35·9%)	19 784 (37·3%)
Multiparous	10 709 (19·8%)	13 346 (25·2%)
Ethnicity*^,^^c^ **(n=108,067)**		
Advantageous	18,205 (33.1%)	17,726 (34.0%)
Disadvantageous	36,784 (66.9%)	35,352 (66.6%)
Gestational Age (mean±SD)*^,^^d^ **(n=101,438)**	**38·5±1·8**	**38·6±2·0**
<37 Weeks	8474 (17.5%)	9424(17.8%)
≥37Weeks	40,048 (82.5%)	43,492 (82.2%)
Birth Weight (mean±SD)*^,^^e^ **(n=107,431)**	**2889·0±507·6**	**2948·5±518·1**
<2500 Grams	6850 (12·7%)	5534 (10·4%)
≥2500 Grams	47 140 (87·3%)	47 907 (89·6%)
BMI(n=4100)*^,^^f^	**24·10±6·4**	**22·3±4·9**
≤18	57 (5·1%)	92 (3·1%)
19−34	1031 (92·7%)	2858 (95·6%)
≥35	24 (2·2%)	38 (1·3%)
Wealth Quintile*^,^^g^**(n=65,045)**		
Quintile 1	5948 (17·8%)	7086 (22·5%)
Quintile 2	6964 (20·8%)	6085 (19·3%)
Quintile 3	6435 (19·2%)	6089 (19·3%)
Quintile 4	5237 (15·6%)	4296 (13·6%)
Quintile 5	8907 (26·6%)	7998 (25·3%)

⁎ Variables with missing information:

a 1447

b 1678

c 836

d 7465

e 1472

f 104,803

g 43,858 Gurung. Effect of meso-and micro-level quality improvement interventions to improve intrapartum care and birth outcome in Nepal- stepped wedge cluster randomized trial. Am J Obstet Gynecol 2026.

The effect of the QI intervention on intrapartum-related mortality varied across 8 hospitals in Nepal. For instance, Seti Provincial Hospital has an IRR of 0.46 (95% CI: 0.31–0.69), indicating a significant reduction in mortality. On the other hand, Dadeldhura Hospital has an IRR of 1.51 (95% CI: 0.75–3.06), suggesting an increase in mortality during the intervention period, however this is non-significant. The pooled effect size across all hospitals is 0.84 (95% CI: 0.66–1.08), indicating a slight reduction in intrapartum-related mortality favoring the intervention (Figure 3).

**Figure 3:**
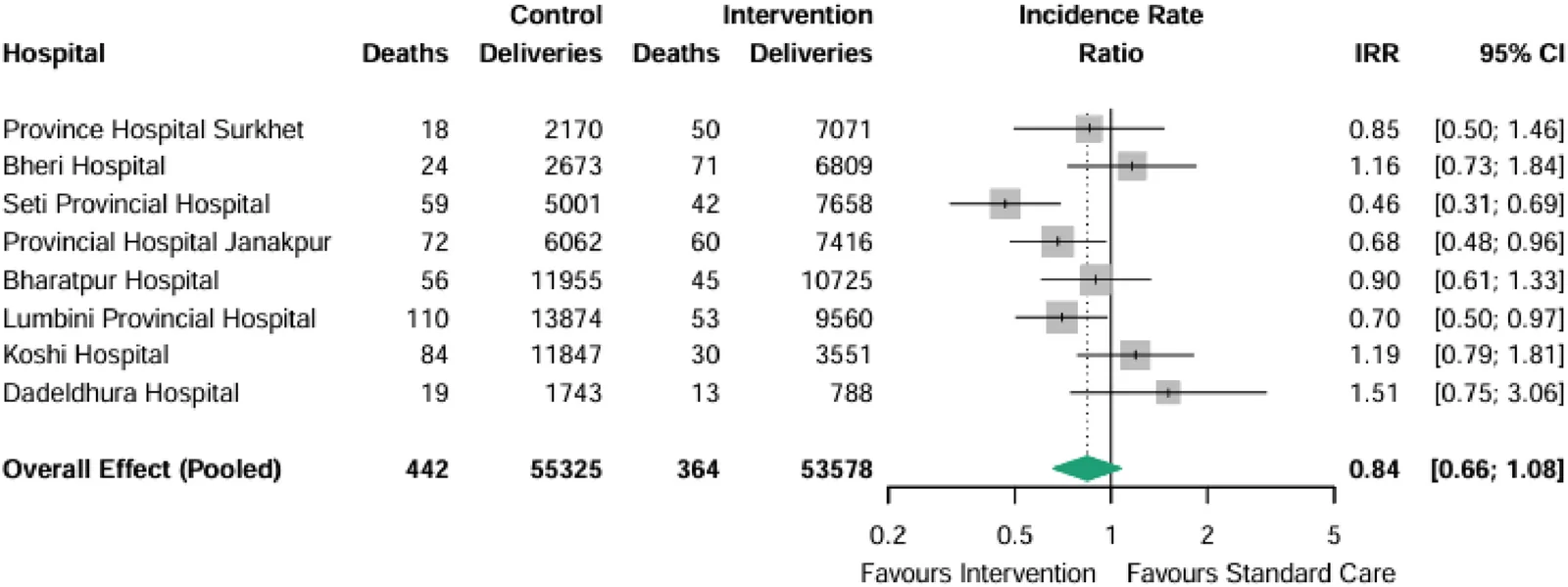
Intrapartum mortality rate by hospital *Gurung. Effect of meso-and micro-level quality improvement interventions to improve intrapartum care and birth outcome in Nepal- stepped wedge cluster randomized trial. Am J Obstet Gynecol 2026.*

At the individual level, when adjusted with all the individual parameters, compared to the pre-intervention period, the odds of intrapartum mortality reduced by 47% (aOR: 0.53, 95% CI: 0.41–0.69, *P*<.0001), indicating a positive effect of the intervention. However, when time adjusted, the odds of intrapartum related mortality reduced non-significantly by 37% (aOR: 0.63, 95% CI, 0.38, 1.04) (Table 3).

**Table 3:** Predictors for intrapartum mortality.

Covariate / Model Parameter	No time-adjusted^a^ aOR (95% CI)	*P*-value	Time-adjusted^b^ aOR (95% CI)	*P*-value
Intervention (After vs Before)	0.53 (0.41–0.69)	<.001	0.63 (0.38–1.04)	.072
Maternal Age (per 1-year increase)	1.03 (1.00–1.05)	.034	1.03 (1.00–1.05)	.041
Ethnicity (Disadvantageous vs Advantageous)	0.95 (0.67–1.35)	.773	0.97 (0.68–1.38)	.858
Maternal Education (Informal vs Formal)	1.91 (1.42–2.57)	<.001	1.91 (1.42–2.57)	<.001
Wealth: Poorer (vs Poorest)	0.83 (0.60–1.14)	.251	0.80 (0.58–1.11)	.190
Wealth: Middle (vs Poorest)	0.65 (0.44–0.94)	.024	0.62 (0.43–0.91)	.016
Wealth: Richer (vs Poorest)	0.57 (0.36–0.88)	.012	0.54 (0.34–0.84)	.007
Wealth: Richest (vs Poorest)	0.36 (0.23–0.55)	<.001	0.34 (0.22–0.52)	<.001
Twin First Baby (vs Single)	3.19 (1.86–5.46)	<.001	3.14 (1.83–5.39)	<.001
Admission Complication (Yes vs No)	3.88 (2.98–5.06)	<.001	3.91 (2.99–5.12)	<.001
Preterm Gestational Age (<37 wks vs ≥37 wks)	5.37 (4.08–7.06)	<.001	5.40 (4.10–7.11)	<.001

Complete-case multivariable models for intrapartum mortality with and without calendar time adjustment.

a Intrapartum Mortality ∼ Intervention + Maternal Age + Ethnicity + Maternal Education + Wealth Quintile + Twin Delivery + Admission Complications + Preterm Birth + (1 | Hospital) (N=58,508; 271 deaths).

b Intrapartum Mortality ∼ Intervention + Calendar Month + Maternal Age + Ethnicity + Maternal Education + Wealth Quintile + Twin Delivery + Admission Complications + Preterm Birth + (1 ∣ Hospital) (N=58,508; 271 deaths). Gurung. Effect of meso-and micro-level quality improvement interventions to improve intrapartum care and birth outcome in Nepal- stepped wedge cluster randomized trial. Am J Obstet Gynecol 2026.

The pooled IRR for handwashing practices and fetal heart rate monitoring was 2.36 (95% CI: 1.03, 5.39), and 1.55 (95% CI: 1.10, 2.17), respectively, indicating significant improvements after the intervention. For delayed cord clamping, the pooled IRR was 1.14 (95% CI: 0.96, 1.35), showing a slight but non-significant improvement after intervention. For the immediate initiation of skin-to-skin contact after birth, the pooled IRR was 9.92 (95% CI: 3.21, 30.64), demonstrating a significant improvement following the intervention. Notably, Lumbini Provincial Hospital has an IRR of 42.47 (95% CI: 32.38, 55.96), highlighting a substantial increase during the intervention period. The pooled IRR for early breastfeeding was 4.09 (95% CI: 2.07, 8.11), indicating a significant improvement practice. Bheri Hospital showed a significant increase with an IRR of 16.64 (95% CI: 12.68, 21.83). Lastly, the pooled IRR for BMV within 1 minute was 3.30 (95% CI: 2.02, 5.40). The overall effect estimation for all secondary outcomes is 2.91 (95% CI: 2.04, 4.13), favoring the SUSTAIN QI package intervention (Figure 4).

**Figure 4:**
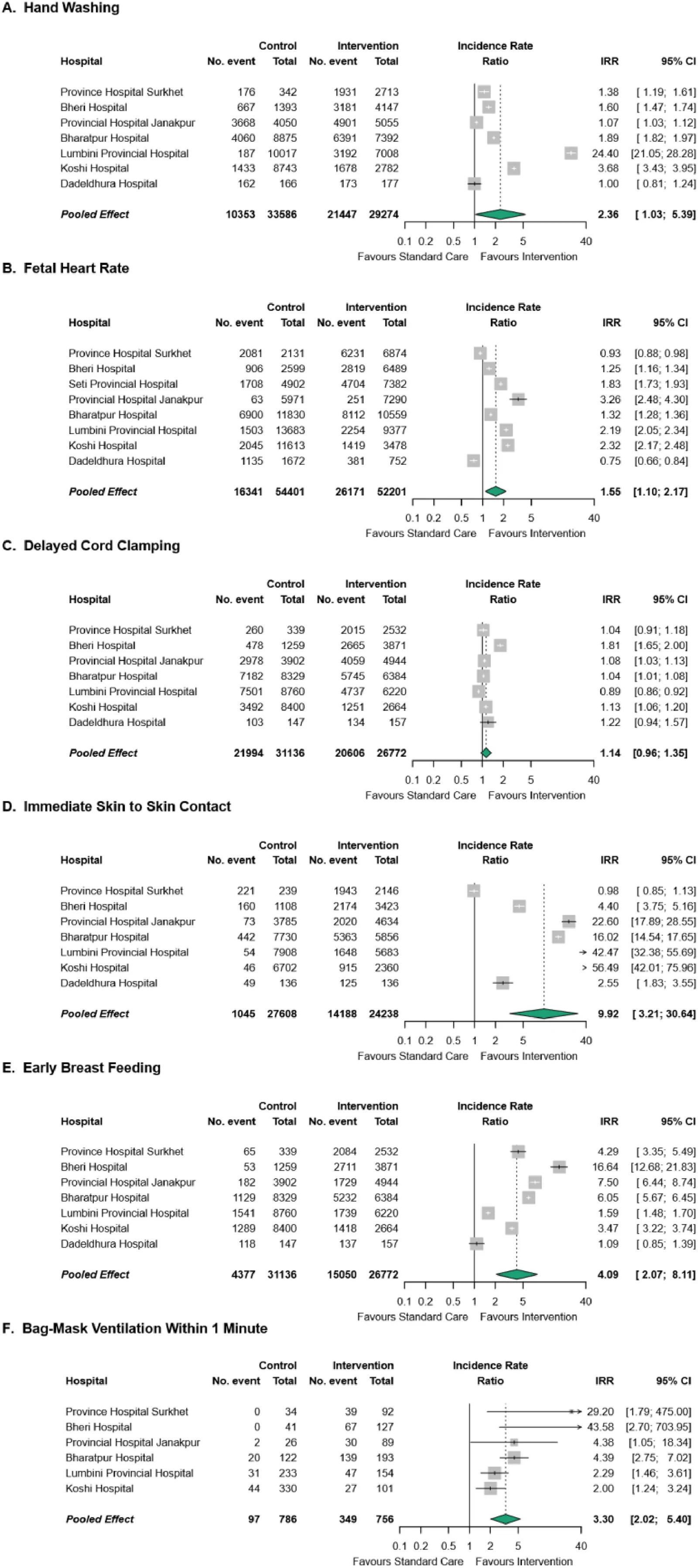
Intervention effect on all secondary outcomes *Gurung. Effect of meso-and micro-level quality improvement interventions to improve intrapartum care and birth outcome in Nepal- stepped wedge cluster randomized trial. Am J Obstet Gynecol 2026.*

The incidence of all outcomes (intrapartum mortality, hand washing, fetal heart rate monitoring, delayed cord clamping, skin-to-skin contact, breastfeeding, and BMV within 1 minute) over time, from April 2019 to February 2021, is shown in Figure 5. The timeline was divided into 3 periods: before the intervention (blue shaded area), during the intervention introduction (white area), and after the intervention was fully introduced (red shaded area). The dashed red line indicates when COVID-19 started in Nepal (around February 2020). The figure showed that intrapartum mortality fluctuated between 0% and 2% during the baseline period and stabilized at slightly lower rates (∼1%) post-intervention, indicating modest improvement. Hand washing adherence, initially low (<50%), increased sharply after the intervention to 60% to 75%, reflecting substantial improvement in hygiene practices. Fetal heart rate monitoring compliance improved from 25% to 45% at baseline to 50% to 75% post-intervention, suggesting enhanced monitoring practices. Delayed cord clamping, which showed a baseline adherence of 25% to 75%, achieved higher and sustained compliance after the intervention. Immediate skin-to-skin contact and early breastfeeding, initially very low (<25% and <15%, respectively), demonstrated marked increases post-intervention, with adherence ranging between 40% and 80%, indicating strong intervention effectiveness. BMV within 1 minute increased clearly after the intervention started, translating into success of the intervention (Figure 5 and Supplementary Table 1).

**Figure 5:**
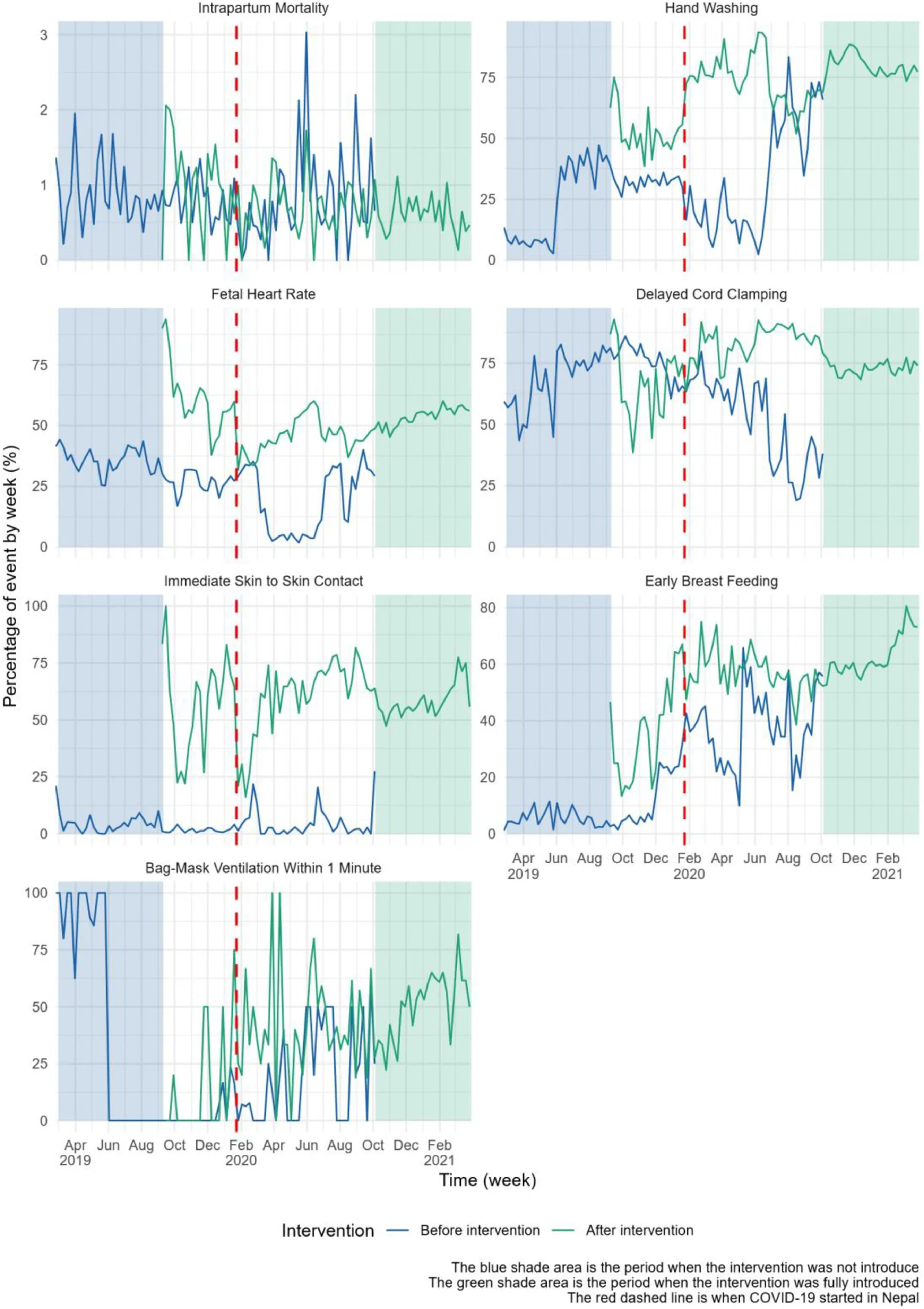
Overall trend of primary and secondary outcomes *Gurung. Effect of meso-and micro-level quality improvement interventions to improve intrapartum care and birth outcome in Nepal- stepped wedge cluster randomized trial. Am J Obstet Gynecol 2026.*

Moreover, the onset of the COVID-19 pandemic coincided with temporary disruptions in some practices. For example, hand washing continued to improve, potentially reflecting heightened hygiene awareness, whereas fetal heart rate monitoring, immediate skin-to-skin contact, and early initiation of breastfeeding experienced transient lows in early 2020 (Figure 5).

## Discussion

This stepped wedge RCT aimed to assess the effect of the intervention on intrapartum related mortality in 8 selected hospitals in Nepal, and to evaluate changes in healthcare providers’ care practices. Overall, the intervention was associated with a non-significant reduction in intrapartum mortality even during the COVID-19 pandemic. For the secondary outcomes, substantial and consistent improvements were observed across key indicators for quality of care. Hand washing practices, fetal heart rate monitoring, initiation of immediate skin-to-skin contact, early initiation of breastfeeding, and BMV within 1 minute increased significantly after intervention implementation. The gain for delayed cord clamping was sustained, but more modest. For the trend, temporary interruptions were observed around the onset of the COVID-19 pandemic, however, improvements in care practices were mostly maintained. Significant predictors included maternal age, informal education, lower wealth quintiles, twin pregnancies, admission complications, and preterm births. The findings highlight the effectiveness of the intervention and the importance of socioeconomic and clinical factors in intrapartum outcomes.

Putting our findings into context, systematic reviews covering more than QI interventions found that while continuous education helps promote compliance with care standards, many strategies targeting individuals, including educational material, audits, and feedback alone, only yield moderate improvements in the quality of care.^22^ Our study adds value by showing that multifaceted interventions, combining meso-level QI intervention implementation with micro-level competency building, can translate into better survival outcomes. The system level support combined with individual level effort can bring changes so that there is better surveillance and care for high-risk mothers and newborns.^23^

Our study has some considerable strengths to highlight. The first explicit strength is the large-scale and real-world application of the SUSTAIN package across diverse hospital setting in Nepal, thereby enhancing applicability to other settings with similar health system bottlenecks. Secondly, despite disruptions due to the onset of the Covid-19 pandemic, improvements in care practices were mostly maintained, which indicates resilience of the intervention in light of public health emergencies. However, this translates into a notable limitation of the intervention; the COVID-19 pandemic prevented the training from being conducted as planned and resulted in delays in the implementation of the interventions. Moreover, starting the intervention with medium-volume hospitals may have affected the exposure outcomes. Another possible limitation of our study was that data collectors may have subjective bias belonging to the cohort of health professionals and clinical training in that hospital. This is because they were locally registered nurses who have been exposed to the hospital routines during their pre-service education; however, they were not directly involved in daily hospital activities.

We suggest that the primary mechanism for the observed improvements is the synergistic nature of the SUSTAIN package. We integrated and modified the WHO Safe Childbirth and Checklist^24^ with facilitated PDSA cycles, thereby empowering the capability to detect local bottlenecks and change provider behavior based on context needs. A study from South India employed a facility- and team-driven approach that proved to be feasible and sustainable while significantly improving indicators such as exclusive breastfeeding and Kangaroo Mother Care.^25^ Our results confirm that system level support and accountability create a favorable environment where healthcare workers can effectively apply their technical caretaking skills. Contrary to our previous study employing the Nepal Perinatal Quality Improvement Project (NePeriQIP),^8^ the SUSTAIN package provided data-driven quality improvement for each hospital individually. In NePeriQIP, during PDSA, bottleneck for improving care were discussed during the meetings with the review of action plan implemented and planning further to overcome the bottleneck. In SUSTAIN during the PDSA meeting, health care providers reviewed the changes in the health care performance in intrapartum care and identified the bottleneck in the measures that lacked progress. Further plans to improve the care were discussed in the PDSA meeting. This likely enabled the close monitoring of process indicators, which may have served as motivation for healthcare professionals by seeing the improvements achieved and thereby contributing to sustainable practice. This has been identified in 1 of our previous qualitative study about the facilitators and barriers of a QI package which included PDSA meetings.^19^ A multi-country study in sub-Saharan Africa showed that implementation of multi-facted QI intervention including PDSA meetings can improve intrapartum care with reduction in early neonatal mortality but not fresh stillbirth.^26^ Similarly, a study from Tanzania^27^ which used similar approach to ours showed reduction in neonatal (up to 24 hours post birth) mortality was observed, while the number of intrapartum stillbirth increased. This may explain the differing findings and be the reason why each program should be supported by continuous data-driven QI initiatives and a strong leadership to achieve the defined targets step-by-step.

This study contributes to discussion regarding the optimal level of macro-level intervention. While the *Lancet Commission on High Quality Health* systems calls for macro-level structural reforms,^6^ our evidence indicates that mixed methods QI strategies at the meso- and micro-level are feasible in a healthcare system that is highly fragmented like Nepal’s.^28^ Our findings suggest focusing on the hospital environment (meso-level) on creating a conducive space for healthcare workers to develop and apply their technical skills.

Future research should focus on the long-term sustainability of the observed survival and other indicator improvements to assess whether changes can be maintained over time.^2^^,^^8^ Additionally, cost evaluations of such QI packages compared to standard practices are needed to get a holistic overview of the QI package and be able to inform policymakers about the economic aspect of the intervention. This study enrolled high-volume hospitals (>3000 deliveries annually) only, necessitating more research into medium- and low-volume settings (with less than 3000 annual deliveries), to assess the QI package as different contexts may require different management approaches. Since this study targeted the micro- and meso-level, more research targeting the macro-level would enhance the implementation research. Zaka et al.^29^ found that out of the 28 studies included in their review about QI initiatives for hospitalized small and sick newborns in low- and middle-income countries, only 2 studies were macro-level interventions. Additionally, the authors found that equity in care delivery was not assessed as an outcome,^29^ and our study did not close this research gap. Since our findings revealed significant differences when considering predictors for mortality, such as age, education and wealth, adding this dimension to QI interventions will possibly yield better outcome, however, further research is needed.

Our findings underscore that achieving SDG 3.2 requires a shift from focusing solely on service access to ensuring a national quality guarantee.^8^ Delivering maternal and newborn health services of low quality is ineffective, wasteful, and deemed unethical.^6^ For policymakers, the SUSTAIN package provides a scalable model that does not require advanced neonatal technology but rather invests in the foundations of the health system: skilled personnel, strengthened leadership, and data-driven clinical processes.

## CRediT authorship contribution statement

**Rejina Gurung:** Writing – review & editing, Validation, Project administration, Methodology, Conceptualization. **Omkar Basnet:** Writing – review & editing, Supervision, Software, Project administration, Formal analysis, Conceptualization. **Prajwal Paudel:** Writing – review & editing, Supervision, Project administration, Methodology, Investigation. **Lea Kreyenbaum:** Writing – review & editing, Writing – original draft, Visualization, Software, Investigation. **Pratiksha Bhattarai:** Writing – review & editing, Visualization, Resources, Project administration, Investigation, Formal analysis. **Abhishek Gurung:** Writing – review & editing, Supervision, Project administration, Formal analysis, Conceptualization. **Honey Malla:** Writing – review & editing, Supervision, Software, Resources, Project administration, Conceptualization. **Avinash K Sunny:** Writing – review & editing, Supervision, Resources, Project administration, Investigation, Formal analysis, Conceptualization. **Srijana Sharma:** Writing – review & editing, Validation, Supervision, Resources, Data curation, Conceptualization. **Anna Axelin:** Writing – review & editing, Visualization, Supervision, Formal analysis, Conceptualization. **Mats Målqvist:** Writing – review & editing, Validation, Supervision, Investigation. **Luong Nguyen Thanh:** Writing – review & editing, Writing – original draft, Visualization, Software, Formal analysis, Data curation. **Ashish Kc:** Writing – review & editing, Visualization, Supervision, Software, Resources, Project administration, Methodology, Conceptualization.

## Appendix Supplementary materials

Image, application 1

Image, application 2

## Footnotes

Supplementary material associated with this article can be found in the online version at doi:10.1016/j.xagr.2026.100697.
