# Supplementary material for: Effect of meso-and micro-level quality improvement interventions to improve intrapartum care and birth outcome in Nepal- stepped wedge cluster randomized trial
Source: AJOG Glob Rep. 2026 Sep 16;6(4):100697. doi: 10.1016/j.xagr.2026.100697 (PMC13677397; doi:10.1016/j.xagr.2026.100697)
Supplement: Supplementary file 2 [file mmc2.docx]

**SUPPLEMENTARY DOCUMENT**

Effect of Meso-and Micro-Level Quality Improvement Interventions to Improve Intrapartum Care and Birth Outcome in Nepal- Stepped Wedge Cluster Randomized Trial

| weekcount | Actual_PeriodITT | total | event | rate | sub_outcome |
| --- | --- | --- | --- | --- | --- |
| 2019-02-24 | Before intervention | 219 | 3 | 1.3698630 | Intrapartum Mortality |
| 2019-03-03 | Before intervention | 876 | 8 | 0.9132420 | Intrapartum Mortality |
| 2019-03-10 | Before intervention | 916 | 2 | 0.2183406 | Intrapartum Mortality |
| 2019-03-17 | Before intervention | 859 | 6 | 0.6984866 | Intrapartum Mortality |
| 2019-03-24 | Before intervention | 780 | 7 | 0.8974359 | Intrapartum Mortality |
| 2019-03-31 | Before intervention | 820 | 16 | 1.9512195 | Intrapartum Mortality |
| 2019-04-07 | Before intervention | 765 | 7 | 0.9150327 | Intrapartum Mortality |
| 2019-04-14 | Before intervention | 655 | 2 | 0.3053435 | Intrapartum Mortality |
| 2019-04-21 | Before intervention | 627 | 5 | 0.7974482 | Intrapartum Mortality |
| 2019-04-28 | Before intervention | 798 | 8 | 1.0025063 | Intrapartum Mortality |
| 2019-05-05 | Before intervention | 838 | 4 | 0.4773270 | Intrapartum Mortality |
| 2019-05-12 | Before intervention | 826 | 11 | 1.3317191 | Intrapartum Mortality |
| 2019-05-19 | Before intervention | 478 | 8 | 1.6736402 | Intrapartum Mortality |
| 2019-05-26 | Before intervention | 383 | 3 | 0.7832898 | Intrapartum Mortality |
| 2019-06-02 | Before intervention | 438 | 3 | 0.6849315 | Intrapartum Mortality |
| 2019-06-09 | Before intervention | 772 | 13 | 1.6839378 | Intrapartum Mortality |
| 2019-06-16 | Before intervention | 927 | 9 | 0.9708738 | Intrapartum Mortality |
| 2019-06-23 | Before intervention | 988 | 6 | 0.6072874 | Intrapartum Mortality |
| 2019-06-30 | Before intervention | 964 | 12 | 1.2448133 | Intrapartum Mortality |
| 2019-07-07 | Before intervention | 994 | 5 | 0.5030181 | Intrapartum Mortality |
| 2019-07-14 | Before intervention | 1,223 | 7 | 0.5723630 | Intrapartum Mortality |
| 2019-07-21 | Before intervention | 1,399 | 12 | 0.8577555 | Intrapartum Mortality |
| 2019-07-28 | Before intervention | 1,471 | 12 | 0.8157716 | Intrapartum Mortality |
| 2019-08-04 | Before intervention | 1,343 | 5 | 0.3723008 | Intrapartum Mortality |
| 2019-08-11 | Before intervention | 1,114 | 9 | 0.8078995 | Intrapartum Mortality |
| 2019-08-18 | Before intervention | 1,321 | 9 | 0.6813020 | Intrapartum Mortality |
| 2019-08-25 | Before intervention | 1,371 | 12 | 0.8752735 | Intrapartum Mortality |
| 2019-09-01 | Before intervention | 1,376 | 9 | 0.6540698 | Intrapartum Mortality |
| 2019-09-08 | Before intervention | 1,186 | 11 | 0.9274874 | Intrapartum Mortality |
| 2019-09-08 | After intervention | 63 | 0 | 0.0000000 | Intrapartum Mortality |
| 2019-09-15 | Before intervention | 1,096 | 8 | 0.7299270 | Intrapartum Mortality |
| 2019-09-15 | After intervention | 97 | 2 | 2.0618557 | Intrapartum Mortality |
| 2019-09-22 | Before intervention | 1,110 | 8 | 0.7207207 | Intrapartum Mortality |
| 2019-09-22 | After intervention | 100 | 2 | 2.0000000 | Intrapartum Mortality |
| 2019-09-29 | Before intervention | 873 | 8 | 0.9163803 | Intrapartum Mortality |
| 2019-09-29 | After intervention | 228 | 4 | 1.7543860 | Intrapartum Mortality |
| 2019-10-06 | Before intervention | 735 | 8 | 1.0884354 | Intrapartum Mortality |
| 2019-10-06 | After intervention | 201 | 2 | 0.9950249 | Intrapartum Mortality |
| 2019-10-13 | Before intervention | 1,015 | 5 | 0.4926108 | Intrapartum Mortality |
| 2019-10-13 | After intervention | 207 | 3 | 1.4492754 | Intrapartum Mortality |
| 2019-10-20 | Before intervention | 1,010 | 8 | 0.7920792 | Intrapartum Mortality |
| 2019-10-20 | After intervention | 182 | 2 | 1.0989011 | Intrapartum Mortality |
| 2019-10-27 | Before intervention | 885 | 11 | 1.2429379 | Intrapartum Mortality |
| 2019-10-27 | After intervention | 149 | 0 | 0.0000000 | Intrapartum Mortality |
| 2019-11-03 | Before intervention | 997 | 5 | 0.5015045 | Intrapartum Mortality |
| 2019-11-03 | After intervention | 185 | 2 | 1.0810811 | Intrapartum Mortality |
| 2019-11-10 | Before intervention | 1,016 | 10 | 0.9842520 | Intrapartum Mortality |
| 2019-11-10 | After intervention | 162 | 2 | 1.2345679 | Intrapartum Mortality |
| 2019-11-17 | Before intervention | 961 | 13 | 1.3527575 | Intrapartum Mortality |
| 2019-11-17 | After intervention | 171 | 1 | 0.5847953 | Intrapartum Mortality |
| 2019-11-24 | Before intervention | 1,064 | 9 | 0.8458647 | Intrapartum Mortality |
| 2019-11-24 | After intervention | 191 | 0 | 0.0000000 | Intrapartum Mortality |
| 2019-12-01 | Before intervention | 1,033 | 10 | 0.9680542 | Intrapartum Mortality |
| 2019-12-01 | After intervention | 212 | 3 | 1.4150943 | Intrapartum Mortality |
| 2019-12-08 | Before intervention | 880 | 3 | 0.3409091 | Intrapartum Mortality |
| 2019-12-08 | After intervention | 375 | 4 | 1.0666667 | Intrapartum Mortality |
| 2019-12-15 | Before intervention | 871 | 5 | 0.5740528 | Intrapartum Mortality |
| 2019-12-15 | After intervention | 454 | 7 | 1.5418502 | Intrapartum Mortality |
| 2019-12-22 | Before intervention | 932 | 5 | 0.5364807 | Intrapartum Mortality |
| 2019-12-22 | After intervention | 390 | 4 | 1.0256410 | Intrapartum Mortality |
| 2019-12-29 | Before intervention | 961 | 9 | 0.9365245 | Intrapartum Mortality |
| 2019-12-29 | After intervention | 335 | 3 | 0.8955224 | Intrapartum Mortality |
| 2020-01-05 | Before intervention | 923 | 5 | 0.5417118 | Intrapartum Mortality |
| 2020-01-05 | After intervention | 356 | 0 | 0.0000000 | Intrapartum Mortality |
| 2020-01-12 | Before intervention | 843 | 4 | 0.4744958 | Intrapartum Mortality |
| 2020-01-12 | After intervention | 299 | 3 | 1.0033445 | Intrapartum Mortality |
| 2020-01-19 | Before intervention | 826 | 9 | 1.0895884 | Intrapartum Mortality |
| 2020-01-19 | After intervention | 320 | 2 | 0.6250000 | Intrapartum Mortality |
| 2020-01-26 | Before intervention | 682 | 3 | 0.4398827 | Intrapartum Mortality |
| 2020-01-26 | After intervention | 503 | 5 | 0.9940358 | Intrapartum Mortality |
| 2020-02-02 | Before intervention | 652 | 0 | 0.0000000 | Intrapartum Mortality |
| 2020-02-02 | After intervention | 494 | 0 | 0.0000000 | Intrapartum Mortality |
| 2020-02-09 | Before intervention | 643 | 1 | 0.1555210 | Intrapartum Mortality |
| 2020-02-09 | After intervention | 477 | 3 | 0.6289308 | Intrapartum Mortality |
| 2020-02-16 | Before intervention | 651 | 5 | 0.7680492 | Intrapartum Mortality |
| 2020-02-16 | After intervention | 497 | 2 | 0.4024145 | Intrapartum Mortality |
| 2020-02-23 | Before intervention | 646 | 3 | 0.4643963 | Intrapartum Mortality |
| 2020-02-23 | After intervention | 500 | 5 | 1.0000000 | Intrapartum Mortality |
| 2020-03-01 | Before intervention | 454 | 2 | 0.4405286 | Intrapartum Mortality |
| 2020-03-01 | After intervention | 489 | 4 | 0.8179959 | Intrapartum Mortality |
| 2020-03-08 | Before intervention | 367 | 1 | 0.2724796 | Intrapartum Mortality |
| 2020-03-08 | After intervention | 595 | 3 | 0.5042017 | Intrapartum Mortality |
| 2020-03-15 | Before intervention | 374 | 3 | 0.8021390 | Intrapartum Mortality |
| 2020-03-15 | After intervention | 625 | 1 | 0.1600000 | Intrapartum Mortality |
| 2020-03-22 | Before intervention | 304 | 0 | 0.0000000 | Intrapartum Mortality |
| 2020-03-22 | After intervention | 435 | 2 | 0.4597701 | Intrapartum Mortality |
| 2020-03-29 | Before intervention | 255 | 2 | 0.7843137 | Intrapartum Mortality |
| 2020-03-29 | After intervention | 515 | 7 | 1.3592233 | Intrapartum Mortality |
| 2020-04-05 | Before intervention | 253 | 1 | 0.3952569 | Intrapartum Mortality |
| 2020-04-05 | After intervention | 460 | 6 | 1.3043478 | Intrapartum Mortality |
| 2020-04-12 | Before intervention | 248 | 3 | 1.2096774 | Intrapartum Mortality |
| 2020-04-12 | After intervention | 403 | 3 | 0.7444169 | Intrapartum Mortality |
| 2020-04-19 | Before intervention | 268 | 3 | 1.1194030 | Intrapartum Mortality |
| 2020-04-19 | After intervention | 396 | 4 | 1.0101010 | Intrapartum Mortality |
| 2020-04-26 | Before intervention | 292 | 2 | 0.6849315 | Intrapartum Mortality |
| 2020-04-26 | After intervention | 464 | 3 | 0.6465517 | Intrapartum Mortality |
| 2020-05-03 | Before intervention | 250 | 1 | 0.4000000 | Intrapartum Mortality |
| 2020-05-03 | After intervention | 426 | 3 | 0.7042254 | Intrapartum Mortality |
| 2020-05-10 | Before intervention | 205 | 1 | 0.4878049 | Intrapartum Mortality |
| 2020-05-10 | After intervention | 392 | 2 | 0.5102041 | Intrapartum Mortality |
| 2020-05-17 | Before intervention | 235 | 5 | 2.1276596 | Intrapartum Mortality |
| 2020-05-17 | After intervention | 350 | 1 | 0.2857143 | Intrapartum Mortality |
| 2020-05-24 | Before intervention | 218 | 2 | 0.9174312 | Intrapartum Mortality |
| 2020-05-24 | After intervention | 368 | 2 | 0.5434783 | Intrapartum Mortality |
| 2020-05-31 | Before intervention | 264 | 8 | 3.0303030 | Intrapartum Mortality |
| 2020-05-31 | After intervention | 406 | 7 | 1.7241379 | Intrapartum Mortality |
| 2020-06-07 | Before intervention | 255 | 2 | 0.7843137 | Intrapartum Mortality |
| 2020-06-07 | After intervention | 414 | 4 | 0.9661836 | Intrapartum Mortality |
| 2020-06-14 | Before intervention | 285 | 4 | 1.4035088 | Intrapartum Mortality |
| 2020-06-14 | After intervention | 447 | 0 | 0.0000000 | Intrapartum Mortality |
| 2020-06-21 | Before intervention | 296 | 2 | 0.6756757 | Intrapartum Mortality |
| 2020-06-21 | After intervention | 496 | 4 | 0.8064516 | Intrapartum Mortality |
| 2020-06-28 | Before intervention | 213 | 1 | 0.4694836 | Intrapartum Mortality |
| 2020-06-28 | After intervention | 821 | 7 | 0.8526188 | Intrapartum Mortality |
| 2020-07-05 | Before intervention | 175 | 1 | 0.5714286 | Intrapartum Mortality |
| 2020-07-05 | After intervention | 904 | 5 | 0.5530973 | Intrapartum Mortality |
| 2020-07-12 | Before intervention | 168 | 2 | 1.1904762 | Intrapartum Mortality |
| 2020-07-12 | After intervention | 982 | 4 | 0.4073320 | Intrapartum Mortality |
| 2020-07-19 | Before intervention | 202 | 2 | 0.9900990 | Intrapartum Mortality |
| 2020-07-19 | After intervention | 1,015 | 6 | 0.5911330 | Intrapartum Mortality |
| 2020-07-26 | Before intervention | 151 | 0 | 0.0000000 | Intrapartum Mortality |
| 2020-07-26 | After intervention | 1,040 | 3 | 0.2884615 | Intrapartum Mortality |
| 2020-08-02 | Before intervention | 64 | 1 | 1.5625000 | Intrapartum Mortality |
| 2020-08-02 | After intervention | 1,002 | 9 | 0.8982036 | Intrapartum Mortality |
| 2020-08-09 | Before intervention | 106 | 1 | 0.9433962 | Intrapartum Mortality |
| 2020-08-09 | After intervention | 856 | 6 | 0.7009346 | Intrapartum Mortality |
| 2020-08-16 | Before intervention | 122 | 0 | 0.0000000 | Intrapartum Mortality |
| 2020-08-16 | After intervention | 673 | 7 | 1.0401189 | Intrapartum Mortality |
| 2020-08-23 | Before intervention | 164 | 1 | 0.6097561 | Intrapartum Mortality |
| 2020-08-23 | After intervention | 812 | 8 | 0.9852217 | Intrapartum Mortality |
| 2020-08-30 | Before intervention | 182 | 4 | 2.1978022 | Intrapartum Mortality |
| 2020-08-30 | After intervention | 877 | 6 | 0.6841505 | Intrapartum Mortality |
| 2020-09-06 | Before intervention | 190 | 2 | 1.0526316 | Intrapartum Mortality |
| 2020-09-06 | After intervention | 880 | 4 | 0.4545455 | Intrapartum Mortality |
| 2020-09-13 | Before intervention | 193 | 1 | 0.5181347 | Intrapartum Mortality |
| 2020-09-13 | After intervention | 947 | 9 | 0.9503696 | Intrapartum Mortality |
| 2020-09-20 | Before intervention | 203 | 1 | 0.4926108 | Intrapartum Mortality |
| 2020-09-20 | After intervention | 999 | 3 | 0.3003003 | Intrapartum Mortality |
| 2020-09-27 | Before intervention | 185 | 3 | 1.6216216 | Intrapartum Mortality |
| 2020-09-27 | After intervention | 903 | 5 | 0.5537099 | Intrapartum Mortality |
| 2020-10-04 | Before intervention | 152 | 1 | 0.6578947 | Intrapartum Mortality |
| 2020-10-04 | After intervention | 935 | 10 | 1.0695187 | Intrapartum Mortality |
| 2020-10-11 | After intervention | 1,039 | 6 | 0.5774783 | Intrapartum Mortality |
| 2020-10-18 | After intervention | 1,096 | 5 | 0.4562044 | Intrapartum Mortality |
| 2020-10-25 | After intervention | 1,065 | 3 | 0.2816901 | Intrapartum Mortality |
| 2020-11-01 | After intervention | 1,103 | 4 | 0.3626473 | Intrapartum Mortality |
| 2020-11-08 | After intervention | 1,121 | 8 | 0.7136485 | Intrapartum Mortality |
| 2020-11-15 | After intervention | 1,075 | 12 | 1.1162791 | Intrapartum Mortality |
| 2020-11-22 | After intervention | 1,249 | 8 | 0.6405124 | Intrapartum Mortality |
| 2020-11-29 | After intervention | 1,162 | 6 | 0.5163511 | Intrapartum Mortality |
| 2020-12-06 | After intervention | 1,082 | 9 | 0.8317930 | Intrapartum Mortality |
| 2020-12-13 | After intervention | 1,076 | 7 | 0.6505576 | Intrapartum Mortality |
| 2020-12-20 | After intervention | 1,059 | 9 | 0.8498584 | Intrapartum Mortality |
| 2020-12-27 | After intervention | 1,134 | 6 | 0.5291005 | Intrapartum Mortality |
| 2021-01-03 | After intervention | 1,172 | 8 | 0.6825939 | Intrapartum Mortality |
| 2021-01-10 | After intervention | 1,097 | 7 | 0.6381039 | Intrapartum Mortality |
| 2021-01-17 | After intervention | 1,093 | 10 | 0.9149131 | Intrapartum Mortality |
| 2021-01-24 | After intervention | 969 | 5 | 0.5159959 | Intrapartum Mortality |
| 2021-01-31 | After intervention | 1,007 | 8 | 0.7944389 | Intrapartum Mortality |
| 2021-02-07 | After intervention | 1,013 | 4 | 0.3948667 | Intrapartum Mortality |
| 2021-02-14 | After intervention | 970 | 10 | 1.0309278 | Intrapartum Mortality |
| 2021-02-21 | After intervention | 1,002 | 6 | 0.5988024 | Intrapartum Mortality |
| 2021-02-28 | After intervention | 777 | 3 | 0.3861004 | Intrapartum Mortality |
| 2021-03-07 | After intervention | 760 | 1 | 0.1315789 | Intrapartum Mortality |
| 2021-03-14 | After intervention | 773 | 5 | 0.6468305 | Intrapartum Mortality |
| 2021-03-21 | After intervention | 780 | 3 | 0.3846154 | Intrapartum Mortality |
| 2021-03-28 | After intervention | 429 | 2 | 0.4662005 | Intrapartum Mortality |
| 2019-02-24 | Before intervention | 96 | 13 | 13.5416667 | Hand Washing |
| 2019-03-03 | Before intervention | 370 | 30 | 8.1081081 | Hand Washing |
| 2019-03-10 | Before intervention | 360 | 24 | 6.6666667 | Hand Washing |
| 2019-03-17 | Before intervention | 379 | 38 | 10.0263852 | Hand Washing |
| 2019-03-24 | Before intervention | 366 | 24 | 6.5573770 | Hand Washing |
| 2019-03-31 | Before intervention | 396 | 31 | 7.8282828 | Hand Washing |
| 2019-04-07 | Before intervention | 303 | 19 | 6.2706271 | Hand Washing |
| 2019-04-14 | Before intervention | 243 | 13 | 5.3497942 | Hand Washing |
| 2019-04-21 | Before intervention | 108 | 9 | 8.3333333 | Hand Washing |
| 2019-04-28 | Before intervention | 331 | 27 | 8.1570997 | Hand Washing |
| 2019-05-05 | Before intervention | 281 | 20 | 7.1174377 | Hand Washing |
| 2019-05-12 | Before intervention | 327 | 29 | 8.8685015 | Hand Washing |
| 2019-05-19 | Before intervention | 164 | 7 | 4.2682927 | Hand Washing |
| 2019-05-26 | Before intervention | 73 | 2 | 2.7397260 | Hand Washing |
| 2019-06-02 | Before intervention | 224 | 54 | 24.1071429 | Hand Washing |
| 2019-06-09 | Before intervention | 460 | 177 | 38.4782609 | Hand Washing |
| 2019-06-16 | Before intervention | 554 | 182 | 32.8519856 | Hand Washing |
| 2019-06-23 | Before intervention | 610 | 260 | 42.6229508 | Hand Washing |
| 2019-06-30 | Before intervention | 727 | 293 | 40.3026135 | Hand Washing |
| 2019-07-07 | Before intervention | 752 | 239 | 31.7819149 | Hand Washing |
| 2019-07-14 | Before intervention | 788 | 339 | 43.0203046 | Hand Washing |
| 2019-07-21 | Before intervention | 844 | 328 | 38.8625592 | Hand Washing |
| 2019-07-28 | Before intervention | 938 | 432 | 46.0554371 | Hand Washing |
| 2019-08-04 | Before intervention | 858 | 330 | 38.4615385 | Hand Washing |
| 2019-08-11 | Before intervention | 720 | 210 | 29.1666667 | Hand Washing |
| 2019-08-18 | Before intervention | 912 | 430 | 47.1491228 | Hand Washing |
| 2019-08-25 | Before intervention | 917 | 371 | 40.4580153 | Hand Washing |
| 2019-09-01 | Before intervention | 888 | 381 | 42.9054054 | Hand Washing |
| 2019-09-08 | Before intervention | 870 | 337 | 38.7356322 | Hand Washing |
| 2019-09-08 | After intervention | 16 | 10 | 62.5000000 | Hand Washing |
| 2019-09-15 | Before intervention | 772 | 256 | 33.1606218 | Hand Washing |
| 2019-09-15 | After intervention | 28 | 21 | 75.0000000 | Hand Washing |
| 2019-09-22 | Before intervention | 732 | 219 | 29.9180328 | Hand Washing |
| 2019-09-22 | After intervention | 38 | 26 | 68.4210526 | Hand Washing |
| 2019-09-29 | Before intervention | 576 | 149 | 25.8680556 | Hand Washing |
| 2019-09-29 | After intervention | 130 | 63 | 48.4615385 | Hand Washing |
| 2019-10-06 | Before intervention | 515 | 177 | 34.3689320 | Hand Washing |
| 2019-10-06 | After intervention | 117 | 58 | 49.5726496 | Hand Washing |
| 2019-10-13 | Before intervention | 679 | 208 | 30.6332842 | Hand Washing |
| 2019-10-13 | After intervention | 110 | 50 | 45.4545455 | Hand Washing |
| 2019-10-20 | Before intervention | 691 | 221 | 31.9826339 | Hand Washing |
| 2019-10-20 | After intervention | 84 | 47 | 55.9523810 | Hand Washing |
| 2019-10-27 | Before intervention | 586 | 163 | 27.8156997 | Hand Washing |
| 2019-10-27 | After intervention | 78 | 36 | 46.1538462 | Hand Washing |
| 2019-11-03 | Before intervention | 669 | 229 | 34.2301943 | Hand Washing |
| 2019-11-03 | After intervention | 102 | 53 | 51.9607843 | Hand Washing |
| 2019-11-10 | Before intervention | 692 | 208 | 30.0578035 | Hand Washing |
| 2019-11-10 | After intervention | 83 | 32 | 38.5542169 | Hand Washing |
| 2019-11-17 | Before intervention | 649 | 227 | 34.9768875 | Hand Washing |
| 2019-11-17 | After intervention | 86 | 54 | 62.7906977 | Hand Washing |
| 2019-11-24 | Before intervention | 668 | 215 | 32.1856287 | Hand Washing |
| 2019-11-24 | After intervention | 90 | 37 | 41.1111111 | Hand Washing |
| 2019-12-01 | Before intervention | 716 | 236 | 32.9608939 | Hand Washing |
| 2019-12-01 | After intervention | 52 | 28 | 53.8461538 | Hand Washing |
| 2019-12-08 | Before intervention | 695 | 213 | 30.6474820 | Hand Washing |
| 2019-12-08 | After intervention | 54 | 28 | 51.8518519 | Hand Washing |
| 2019-12-15 | Before intervention | 678 | 244 | 35.9882006 | Hand Washing |
| 2019-12-15 | After intervention | 60 | 28 | 46.6666667 | Hand Washing |
| 2019-12-22 | Before intervention | 675 | 208 | 30.8148148 | Hand Washing |
| 2019-12-22 | After intervention | 64 | 31 | 48.4375000 | Hand Washing |
| 2019-12-29 | Before intervention | 696 | 228 | 32.7586207 | Hand Washing |
| 2019-12-29 | After intervention | 44 | 20 | 45.4545455 | Hand Washing |
| 2020-01-05 | Before intervention | 640 | 215 | 33.5937500 | Hand Washing |
| 2020-01-05 | After intervention | 63 | 32 | 50.7936508 | Hand Washing |
| 2020-01-12 | Before intervention | 607 | 209 | 34.4316310 | Hand Washing |
| 2020-01-12 | After intervention | 72 | 39 | 54.1666667 | Hand Washing |
| 2020-01-19 | Before intervention | 550 | 165 | 30.0000000 | Hand Washing |
| 2020-01-19 | After intervention | 81 | 45 | 55.5555556 | Hand Washing |
| 2020-01-26 | Before intervention | 447 | 74 | 16.5548098 | Hand Washing |
| 2020-01-26 | After intervention | 207 | 150 | 72.4637681 | Hand Washing |
| 2020-02-02 | Before intervention | 484 | 148 | 30.5785124 | Hand Washing |
| 2020-02-02 | After intervention | 221 | 167 | 75.5656109 | Hand Washing |
| 2020-02-09 | Before intervention | 432 | 85 | 19.6759259 | Hand Washing |
| 2020-02-09 | After intervention | 207 | 156 | 75.3623188 | Hand Washing |
| 2020-02-16 | Before intervention | 433 | 68 | 15.7043880 | Hand Washing |
| 2020-02-16 | After intervention | 203 | 148 | 72.9064039 | Hand Washing |
| 2020-02-23 | Before intervention | 440 | 60 | 13.6363636 | Hand Washing |
| 2020-02-23 | After intervention | 211 | 172 | 81.5165877 | Hand Washing |
| 2020-03-01 | Before intervention | 314 | 78 | 24.8407643 | Hand Washing |
| 2020-03-01 | After intervention | 298 | 226 | 75.8389262 | Hand Washing |
| 2020-03-08 | Before intervention | 249 | 23 | 9.2369478 | Hand Washing |
| 2020-03-08 | After intervention | 313 | 237 | 75.7188498 | Hand Washing |
| 2020-03-15 | Before intervention | 242 | 13 | 5.3719008 | Hand Washing |
| 2020-03-15 | After intervention | 280 | 210 | 75.0000000 | Hand Washing |
| 2020-03-22 | Before intervention | 125 | 15 | 12.0000000 | Hand Washing |
| 2020-03-22 | After intervention | 103 | 86 | 83.4951456 | Hand Washing |
| 2020-03-29 | Before intervention | 44 | 11 | 25.0000000 | Hand Washing |
| 2020-03-29 | After intervention | 37 | 29 | 78.3783784 | Hand Washing |
| 2020-04-05 | Before intervention | 83 | 28 | 33.7349398 | Hand Washing |
| 2020-04-05 | After intervention | 76 | 69 | 90.7894737 | Hand Washing |
| 2020-04-12 | Before intervention | 78 | 14 | 17.9487179 | Hand Washing |
| 2020-04-12 | After intervention | 86 | 66 | 76.7441860 | Hand Washing |
| 2020-04-19 | Before intervention | 86 | 13 | 15.1162791 | Hand Washing |
| 2020-04-19 | After intervention | 116 | 90 | 77.5862069 | Hand Washing |
| 2020-04-26 | Before intervention | 115 | 19 | 16.5217391 | Hand Washing |
| 2020-04-26 | After intervention | 147 | 105 | 71.4285714 | Hand Washing |
| 2020-05-03 | Before intervention | 87 | 6 | 6.8965517 | Hand Washing |
| 2020-05-03 | After intervention | 169 | 115 | 68.0473373 | Hand Washing |
| 2020-05-10 | Before intervention | 91 | 15 | 16.4835165 | Hand Washing |
| 2020-05-10 | After intervention | 171 | 125 | 73.0994152 | Hand Washing |
| 2020-05-17 | Before intervention | 88 | 14 | 15.9090909 | Hand Washing |
| 2020-05-17 | After intervention | 153 | 116 | 75.8169935 | Hand Washing |
| 2020-05-24 | Before intervention | 73 | 9 | 12.3287671 | Hand Washing |
| 2020-05-24 | After intervention | 160 | 138 | 86.2500000 | Hand Washing |
| 2020-05-31 | Before intervention | 113 | 9 | 7.9646018 | Hand Washing |
| 2020-05-31 | After intervention | 153 | 130 | 84.9673203 | Hand Washing |
| 2020-06-07 | Before intervention | 167 | 4 | 2.3952096 | Hand Washing |
| 2020-06-07 | After intervention | 212 | 198 | 93.3962264 | Hand Washing |
| 2020-06-14 | Before intervention | 191 | 18 | 9.4240838 | Hand Washing |
| 2020-06-14 | After intervention | 267 | 249 | 93.2584270 | Hand Washing |
| 2020-06-21 | Before intervention | 205 | 48 | 23.4146341 | Hand Washing |
| 2020-06-21 | After intervention | 343 | 313 | 91.2536443 | Hand Washing |
| 2020-06-28 | Before intervention | 130 | 53 | 40.7692308 | Hand Washing |
| 2020-06-28 | After intervention | 519 | 353 | 68.0154143 | Hand Washing |
| 2020-07-05 | Before intervention | 118 | 74 | 62.7118644 | Hand Washing |
| 2020-07-05 | After intervention | 603 | 373 | 61.8573798 | Hand Washing |
| 2020-07-12 | Before intervention | 117 | 54 | 46.1538462 | Hand Washing |
| 2020-07-12 | After intervention | 591 | 400 | 67.6818951 | Hand Washing |
| 2020-07-19 | Before intervention | 125 | 67 | 53.6000000 | Hand Washing |
| 2020-07-19 | After intervention | 666 | 445 | 66.8168168 | Hand Washing |
| 2020-07-26 | Before intervention | 101 | 58 | 57.4257426 | Hand Washing |
| 2020-07-26 | After intervention | 671 | 455 | 67.8092399 | Hand Washing |
| 2020-08-02 | Before intervention | 24 | 20 | 83.3333333 | Hand Washing |
| 2020-08-02 | After intervention | 614 | 364 | 59.2833876 | Hand Washing |
| 2020-08-09 | Before intervention | 67 | 42 | 62.6865672 | Hand Washing |
| 2020-08-09 | After intervention | 540 | 328 | 60.7407407 | Hand Washing |
| 2020-08-16 | Before intervention | 81 | 48 | 59.2592593 | Hand Washing |
| 2020-08-16 | After intervention | 447 | 232 | 51.9015660 | Hand Washing |
| 2020-08-23 | Before intervention | 108 | 54 | 50.0000000 | Hand Washing |
| 2020-08-23 | After intervention | 502 | 307 | 61.1553785 | Hand Washing |
| 2020-08-30 | Before intervention | 127 | 44 | 34.6456693 | Hand Washing |
| 2020-08-30 | After intervention | 561 | 341 | 60.7843137 | Hand Washing |
| 2020-09-06 | Before intervention | 127 | 58 | 45.6692913 | Hand Washing |
| 2020-09-06 | After intervention | 514 | 358 | 69.6498054 | Hand Washing |
| 2020-09-13 | Before intervention | 125 | 91 | 72.8000000 | Hand Washing |
| 2020-09-13 | After intervention | 563 | 374 | 66.4298401 | Hand Washing |
| 2020-09-20 | Before intervention | 139 | 93 | 66.9064748 | Hand Washing |
| 2020-09-20 | After intervention | 659 | 454 | 68.8922610 | Hand Washing |
| 2020-09-27 | Before intervention | 149 | 109 | 73.1543624 | Hand Washing |
| 2020-09-27 | After intervention | 587 | 410 | 69.8466780 | Hand Washing |
| 2020-10-04 | Before intervention | 117 | 77 | 65.8119658 | Hand Washing |
| 2020-10-04 | After intervention | 624 | 430 | 68.9102564 | Hand Washing |
| 2020-10-11 | After intervention | 739 | 570 | 77.1312585 | Hand Washing |
| 2020-10-18 | After intervention | 787 | 677 | 86.0228717 | Hand Washing |
| 2020-10-25 | After intervention | 766 | 624 | 81.4621410 | Hand Washing |
| 2020-11-01 | After intervention | 724 | 581 | 80.2486188 | Hand Washing |
| 2020-11-08 | After intervention | 763 | 630 | 82.5688073 | Hand Washing |
| 2020-11-15 | After intervention | 817 | 702 | 85.9241126 | Hand Washing |
| 2020-11-22 | After intervention | 900 | 796 | 88.4444444 | Hand Washing |
| 2020-11-29 | After intervention | 857 | 755 | 88.0980163 | Hand Washing |
| 2020-12-06 | After intervention | 788 | 682 | 86.5482234 | Hand Washing |
| 2020-12-13 | After intervention | 808 | 668 | 82.6732673 | Hand Washing |
| 2020-12-20 | After intervention | 816 | 662 | 81.1274510 | Hand Washing |
| 2020-12-27 | After intervention | 802 | 630 | 78.5536160 | Hand Washing |
| 2021-01-03 | After intervention | 834 | 639 | 76.6187050 | Hand Washing |
| 2021-01-10 | After intervention | 805 | 606 | 75.2795031 | Hand Washing |
| 2021-01-17 | After intervention | 754 | 598 | 79.3103448 | Hand Washing |
| 2021-01-24 | After intervention | 664 | 511 | 76.9578313 | Hand Washing |
| 2021-01-31 | After intervention | 699 | 525 | 75.1072961 | Hand Washing |
| 2021-02-07 | After intervention | 692 | 530 | 76.5895954 | Hand Washing |
| 2021-02-14 | After intervention | 631 | 482 | 76.3866878 | Hand Washing |
| 2021-02-21 | After intervention | 638 | 507 | 79.4670846 | Hand Washing |
| 2021-02-28 | After intervention | 571 | 458 | 80.2101576 | Hand Washing |
| 2021-03-07 | After intervention | 556 | 408 | 73.3812950 | Hand Washing |
| 2021-03-14 | After intervention | 530 | 407 | 76.7924528 | Hand Washing |
| 2021-03-21 | After intervention | 561 | 448 | 79.8573975 | Hand Washing |
| 2021-03-28 | After intervention | 294 | 227 | 77.2108844 | Hand Washing |
| 2019-02-24 | Before intervention | 215 | 89 | 41.3953488 | Fetal Heart Rate |
| 2019-03-03 | Before intervention | 864 | 382 | 44.2129630 | Fetal Heart Rate |
| 2019-03-10 | Before intervention | 907 | 374 | 41.2348401 | Fetal Heart Rate |
| 2019-03-17 | Before intervention | 853 | 305 | 35.7561547 | Fetal Heart Rate |
| 2019-03-24 | Before intervention | 771 | 293 | 38.0025940 | Fetal Heart Rate |
| 2019-03-31 | Before intervention | 806 | 272 | 33.7468983 | Fetal Heart Rate |
| 2019-04-07 | Before intervention | 758 | 236 | 31.1345646 | Fetal Heart Rate |
| 2019-04-14 | Before intervention | 649 | 224 | 34.5146379 | Fetal Heart Rate |
| 2019-04-21 | Before intervention | 622 | 233 | 37.4598071 | Fetal Heart Rate |
| 2019-04-28 | Before intervention | 788 | 317 | 40.2284264 | Fetal Heart Rate |
| 2019-05-05 | Before intervention | 832 | 293 | 35.2163462 | Fetal Heart Rate |
| 2019-05-12 | Before intervention | 819 | 289 | 35.2869353 | Fetal Heart Rate |
| 2019-05-19 | Before intervention | 473 | 121 | 25.5813953 | Fetal Heart Rate |
| 2019-05-26 | Before intervention | 376 | 95 | 25.2659574 | Fetal Heart Rate |
| 2019-06-02 | Before intervention | 422 | 152 | 36.0189573 | Fetal Heart Rate |
| 2019-06-09 | Before intervention | 750 | 236 | 31.4666667 | Fetal Heart Rate |
| 2019-06-16 | Before intervention | 913 | 298 | 32.6396495 | Fetal Heart Rate |
| 2019-06-23 | Before intervention | 970 | 347 | 35.7731959 | Fetal Heart Rate |
| 2019-06-30 | Before intervention | 949 | 354 | 37.3024236 | Fetal Heart Rate |
| 2019-07-07 | Before intervention | 981 | 412 | 41.9979613 | Fetal Heart Rate |
| 2019-07-14 | Before intervention | 1,208 | 494 | 40.8940397 | Fetal Heart Rate |
| 2019-07-21 | Before intervention | 1,372 | 558 | 40.6705539 | Fetal Heart Rate |
| 2019-07-28 | Before intervention | 1,450 | 538 | 37.1034483 | Fetal Heart Rate |
| 2019-08-04 | Before intervention | 1,316 | 572 | 43.4650456 | Fetal Heart Rate |
| 2019-08-11 | Before intervention | 1,100 | 385 | 35.0000000 | Fetal Heart Rate |
| 2019-08-18 | Before intervention | 1,302 | 387 | 29.7235023 | Fetal Heart Rate |
| 2019-08-25 | Before intervention | 1,346 | 409 | 30.3863299 | Fetal Heart Rate |
| 2019-09-01 | Before intervention | 1,357 | 495 | 36.4775239 | Fetal Heart Rate |
| 2019-09-08 | Before intervention | 1,166 | 352 | 30.1886792 | Fetal Heart Rate |
| 2019-09-08 | After intervention | 60 | 54 | 90.0000000 | Fetal Heart Rate |
| 2019-09-15 | Before intervention | 1,070 | 297 | 27.7570093 | Fetal Heart Rate |
| 2019-09-15 | After intervention | 96 | 90 | 93.7500000 | Fetal Heart Rate |
| 2019-09-22 | Before intervention | 1,087 | 290 | 26.6789328 | Fetal Heart Rate |
| 2019-09-22 | After intervention | 97 | 79 | 81.4432990 | Fetal Heart Rate |
| 2019-09-29 | Before intervention | 856 | 228 | 26.6355140 | Fetal Heart Rate |
| 2019-09-29 | After intervention | 222 | 137 | 61.7117117 | Fetal Heart Rate |
| 2019-10-06 | Before intervention | 724 | 122 | 16.8508287 | Fetal Heart Rate |
| 2019-10-06 | After intervention | 199 | 134 | 67.3366834 | Fetal Heart Rate |
| 2019-10-13 | Before intervention | 1,001 | 213 | 21.2787213 | Fetal Heart Rate |
| 2019-10-13 | After intervention | 198 | 125 | 63.1313131 | Fetal Heart Rate |
| 2019-10-20 | Before intervention | 991 | 315 | 31.7860747 | Fetal Heart Rate |
| 2019-10-20 | After intervention | 173 | 92 | 53.1791908 | Fetal Heart Rate |
| 2019-10-27 | Before intervention | 875 | 279 | 31.8857143 | Fetal Heart Rate |
| 2019-10-27 | After intervention | 147 | 83 | 56.4625850 | Fetal Heart Rate |
| 2019-11-03 | Before intervention | 977 | 311 | 31.8321392 | Fetal Heart Rate |
| 2019-11-03 | After intervention | 178 | 98 | 55.0561798 | Fetal Heart Rate |
| 2019-11-10 | Before intervention | 996 | 313 | 31.4257028 | Fetal Heart Rate |
| 2019-11-10 | After intervention | 149 | 91 | 61.0738255 | Fetal Heart Rate |
| 2019-11-17 | Before intervention | 942 | 237 | 25.1592357 | Fetal Heart Rate |
| 2019-11-17 | After intervention | 162 | 106 | 65.4320988 | Fetal Heart Rate |
| 2019-11-24 | Before intervention | 1,049 | 245 | 23.3555767 | Fetal Heart Rate |
| 2019-11-24 | After intervention | 183 | 117 | 63.9344262 | Fetal Heart Rate |
| 2019-12-01 | Before intervention | 1,021 | 236 | 23.1145935 | Fetal Heart Rate |
| 2019-12-01 | After intervention | 204 | 121 | 59.3137255 | Fetal Heart Rate |
| 2019-12-08 | Before intervention | 861 | 248 | 28.8037166 | Fetal Heart Rate |
| 2019-12-08 | After intervention | 364 | 138 | 37.9120879 | Fetal Heart Rate |
| 2019-12-15 | Before intervention | 861 | 233 | 27.0615563 | Fetal Heart Rate |
| 2019-12-15 | After intervention | 444 | 192 | 43.2432432 | Fetal Heart Rate |
| 2019-12-22 | Before intervention | 918 | 185 | 20.1525054 | Fetal Heart Rate |
| 2019-12-22 | After intervention | 379 | 173 | 45.6464380 | Fetal Heart Rate |
| 2019-12-29 | Before intervention | 950 | 238 | 25.0526316 | Fetal Heart Rate |
| 2019-12-29 | After intervention | 322 | 179 | 55.5900621 | Fetal Heart Rate |
| 2020-01-05 | Before intervention | 913 | 245 | 26.8346112 | Fetal Heart Rate |
| 2020-01-05 | After intervention | 348 | 194 | 55.7471264 | Fetal Heart Rate |
| 2020-01-12 | Before intervention | 836 | 244 | 29.1866029 | Fetal Heart Rate |
| 2020-01-12 | After intervention | 281 | 160 | 56.9395018 | Fetal Heart Rate |
| 2020-01-19 | Before intervention | 812 | 221 | 27.2167488 | Fetal Heart Rate |
| 2020-01-19 | After intervention | 309 | 185 | 59.8705502 | Fetal Heart Rate |
| 2020-01-26 | Before intervention | 673 | 196 | 29.1233284 | Fetal Heart Rate |
| 2020-01-26 | After intervention | 492 | 156 | 31.7073171 | Fetal Heart Rate |
| 2020-02-02 | Before intervention | 647 | 204 | 31.5301391 | Fetal Heart Rate |
| 2020-02-02 | After intervention | 483 | 203 | 42.0289855 | Fetal Heart Rate |
| 2020-02-09 | Before intervention | 637 | 216 | 33.9089482 | Fetal Heart Rate |
| 2020-02-09 | After intervention | 467 | 179 | 38.3297645 | Fetal Heart Rate |
| 2020-02-16 | Before intervention | 645 | 216 | 33.4883721 | Fetal Heart Rate |
| 2020-02-16 | After intervention | 482 | 167 | 34.6473029 | Fetal Heart Rate |
| 2020-02-23 | Before intervention | 639 | 225 | 35.2112676 | Fetal Heart Rate |
| 2020-02-23 | After intervention | 490 | 164 | 33.4693878 | Fetal Heart Rate |
| 2020-03-01 | Before intervention | 446 | 143 | 32.0627803 | Fetal Heart Rate |
| 2020-03-01 | After intervention | 481 | 170 | 35.3430353 | Fetal Heart Rate |
| 2020-03-08 | Before intervention | 362 | 51 | 14.0883978 | Fetal Heart Rate |
| 2020-03-08 | After intervention | 582 | 248 | 42.6116838 | Fetal Heart Rate |
| 2020-03-15 | Before intervention | 368 | 58 | 15.7608696 | Fetal Heart Rate |
| 2020-03-15 | After intervention | 617 | 252 | 40.8427877 | Fetal Heart Rate |
| 2020-03-22 | Before intervention | 302 | 16 | 5.2980132 | Fetal Heart Rate |
| 2020-03-22 | After intervention | 426 | 187 | 43.8967136 | Fetal Heart Rate |
| 2020-03-29 | Before intervention | 244 | 6 | 2.4590164 | Fetal Heart Rate |
| 2020-03-29 | After intervention | 504 | 216 | 42.8571429 | Fetal Heart Rate |
| 2020-04-05 | Before intervention | 238 | 8 | 3.3613445 | Fetal Heart Rate |
| 2020-04-05 | After intervention | 451 | 192 | 42.5720621 | Fetal Heart Rate |
| 2020-04-12 | Before intervention | 238 | 11 | 4.6218487 | Fetal Heart Rate |
| 2020-04-12 | After intervention | 383 | 179 | 46.7362924 | Fetal Heart Rate |
| 2020-04-19 | Before intervention | 259 | 13 | 5.0193050 | Fetal Heart Rate |
| 2020-04-19 | After intervention | 391 | 184 | 47.0588235 | Fetal Heart Rate |
| 2020-04-26 | Before intervention | 286 | 8 | 2.7972028 | Fetal Heart Rate |
| 2020-04-26 | After intervention | 449 | 216 | 48.1069042 | Fetal Heart Rate |
| 2020-05-03 | Before intervention | 247 | 14 | 5.6680162 | Fetal Heart Rate |
| 2020-05-03 | After intervention | 415 | 180 | 43.3734940 | Fetal Heart Rate |
| 2020-05-10 | Before intervention | 199 | 7 | 3.5175879 | Fetal Heart Rate |
| 2020-05-10 | After intervention | 378 | 200 | 52.9100529 | Fetal Heart Rate |
| 2020-05-17 | Before intervention | 229 | 4 | 1.7467249 | Fetal Heart Rate |
| 2020-05-17 | After intervention | 343 | 184 | 53.6443149 | Fetal Heart Rate |
| 2020-05-24 | Before intervention | 211 | 11 | 5.2132701 | Fetal Heart Rate |
| 2020-05-24 | After intervention | 358 | 198 | 55.3072626 | Fetal Heart Rate |
| 2020-05-31 | Before intervention | 257 | 12 | 4.6692607 | Fetal Heart Rate |
| 2020-05-31 | After intervention | 391 | 221 | 56.5217391 | Fetal Heart Rate |
| 2020-06-07 | Before intervention | 246 | 9 | 3.6585366 | Fetal Heart Rate |
| 2020-06-07 | After intervention | 399 | 235 | 58.8972431 | Fetal Heart Rate |
| 2020-06-14 | Before intervention | 275 | 10 | 3.6363636 | Fetal Heart Rate |
| 2020-06-14 | After intervention | 436 | 262 | 60.0917431 | Fetal Heart Rate |
| 2020-06-21 | Before intervention | 287 | 25 | 8.7108014 | Fetal Heart Rate |
| 2020-06-21 | After intervention | 472 | 272 | 57.6271186 | Fetal Heart Rate |
| 2020-06-28 | Before intervention | 203 | 23 | 11.3300493 | Fetal Heart Rate |
| 2020-06-28 | After intervention | 796 | 364 | 45.7286432 | Fetal Heart Rate |
| 2020-07-05 | Before intervention | 169 | 49 | 28.9940828 | Fetal Heart Rate |
| 2020-07-05 | After intervention | 875 | 380 | 43.4285714 | Fetal Heart Rate |
| 2020-07-12 | Before intervention | 161 | 48 | 29.8136646 | Fetal Heart Rate |
| 2020-07-12 | After intervention | 957 | 470 | 49.1118077 | Fetal Heart Rate |
| 2020-07-19 | Before intervention | 195 | 65 | 33.3333333 | Fetal Heart Rate |
| 2020-07-19 | After intervention | 995 | 461 | 46.3316583 | Fetal Heart Rate |
| 2020-07-26 | Before intervention | 147 | 48 | 32.6530612 | Fetal Heart Rate |
| 2020-07-26 | After intervention | 1,014 | 471 | 46.4497041 | Fetal Heart Rate |
| 2020-08-02 | Before intervention | 61 | 21 | 34.4262295 | Fetal Heart Rate |
| 2020-08-02 | After intervention | 979 | 486 | 49.6424923 | Fetal Heart Rate |
| 2020-08-09 | Before intervention | 104 | 12 | 11.5384615 | Fetal Heart Rate |
| 2020-08-09 | After intervention | 831 | 385 | 46.3297232 | Fetal Heart Rate |
| 2020-08-16 | Before intervention | 117 | 12 | 10.2564103 | Fetal Heart Rate |
| 2020-08-16 | After intervention | 657 | 242 | 36.8340944 | Fetal Heart Rate |
| 2020-08-23 | Before intervention | 162 | 47 | 29.0123457 | Fetal Heart Rate |
| 2020-08-23 | After intervention | 787 | 320 | 40.6607370 | Fetal Heart Rate |
| 2020-08-30 | Before intervention | 172 | 41 | 23.8372093 | Fetal Heart Rate |
| 2020-08-30 | After intervention | 853 | 376 | 44.0797186 | Fetal Heart Rate |
| 2020-09-06 | Before intervention | 187 | 61 | 32.6203209 | Fetal Heart Rate |
| 2020-09-06 | After intervention | 855 | 373 | 43.6257310 | Fetal Heart Rate |
| 2020-09-13 | Before intervention | 190 | 76 | 40.0000000 | Fetal Heart Rate |
| 2020-09-13 | After intervention | 920 | 403 | 43.8043478 | Fetal Heart Rate |
| 2020-09-20 | Before intervention | 195 | 63 | 32.3076923 | Fetal Heart Rate |
| 2020-09-20 | After intervention | 973 | 446 | 45.8376156 | Fetal Heart Rate |
| 2020-09-27 | Before intervention | 181 | 57 | 31.4917127 | Fetal Heart Rate |
| 2020-09-27 | After intervention | 881 | 421 | 47.7866061 | Fetal Heart Rate |
| 2020-10-04 | Before intervention | 147 | 43 | 29.2517007 | Fetal Heart Rate |
| 2020-10-04 | After intervention | 915 | 445 | 48.6338798 | Fetal Heart Rate |
| 2020-10-11 | After intervention | 1,011 | 520 | 51.4342235 | Fetal Heart Rate |
| 2020-10-18 | After intervention | 1,064 | 500 | 46.9924812 | Fetal Heart Rate |
| 2020-10-25 | After intervention | 1,045 | 535 | 51.1961722 | Fetal Heart Rate |
| 2020-11-01 | After intervention | 1,072 | 485 | 45.2425373 | Fetal Heart Rate |
| 2020-11-08 | After intervention | 1,079 | 523 | 48.4708063 | Fetal Heart Rate |
| 2020-11-15 | After intervention | 1,048 | 522 | 49.8091603 | Fetal Heart Rate |
| 2020-11-22 | After intervention | 1,216 | 643 | 52.8782895 | Fetal Heart Rate |
| 2020-11-29 | After intervention | 1,135 | 606 | 53.3920705 | Fetal Heart Rate |
| 2020-12-06 | After intervention | 1,057 | 543 | 51.3718070 | Fetal Heart Rate |
| 2020-12-13 | After intervention | 1,056 | 544 | 51.5151515 | Fetal Heart Rate |
| 2020-12-20 | After intervention | 1,037 | 571 | 55.0626808 | Fetal Heart Rate |
| 2020-12-27 | After intervention | 1,113 | 618 | 55.5256065 | Fetal Heart Rate |
| 2021-01-03 | After intervention | 1,150 | 640 | 55.6521739 | Fetal Heart Rate |
| 2021-01-10 | After intervention | 1,064 | 578 | 54.3233083 | Fetal Heart Rate |
| 2021-01-17 | After intervention | 1,066 | 593 | 55.6285178 | Fetal Heart Rate |
| 2021-01-24 | After intervention | 951 | 500 | 52.5762355 | Fetal Heart Rate |
| 2021-01-31 | After intervention | 979 | 546 | 55.7711951 | Fetal Heart Rate |
| 2021-02-07 | After intervention | 998 | 600 | 60.1202405 | Fetal Heart Rate |
| 2021-02-14 | After intervention | 951 | 538 | 56.5720294 | Fetal Heart Rate |
| 2021-02-21 | After intervention | 974 | 556 | 57.0841889 | Fetal Heart Rate |
| 2021-02-28 | After intervention | 765 | 417 | 54.5098039 | Fetal Heart Rate |
| 2021-03-07 | After intervention | 741 | 429 | 57.8947368 | Fetal Heart Rate |
| 2021-03-14 | After intervention | 759 | 443 | 58.3662714 | Fetal Heart Rate |
| 2021-03-21 | After intervention | 760 | 431 | 56.7105263 | Fetal Heart Rate |
| 2021-03-28 | After intervention | 417 | 234 | 56.1151079 | Fetal Heart Rate |
| 2019-02-24 | Before intervention | 86 | 51 | 59.3023256 | Delayed Cord Clamping |
| 2019-03-03 | Before intervention | 325 | 185 | 56.9230769 | Delayed Cord Clamping |
| 2019-03-10 | Before intervention | 322 | 188 | 58.3850932 | Delayed Cord Clamping |
| 2019-03-17 | Before intervention | 317 | 196 | 61.8296530 | Delayed Cord Clamping |
| 2019-03-24 | Before intervention | 311 | 135 | 43.4083601 | Delayed Cord Clamping |
| 2019-03-31 | Before intervention | 350 | 175 | 50.0000000 | Delayed Cord Clamping |
| 2019-04-07 | Before intervention | 270 | 131 | 48.5185185 | Delayed Cord Clamping |
| 2019-04-14 | Before intervention | 220 | 134 | 60.9090909 | Delayed Cord Clamping |
| 2019-04-21 | Before intervention | 91 | 71 | 78.0219780 | Delayed Cord Clamping |
| 2019-04-28 | Before intervention | 299 | 193 | 64.5484950 | Delayed Cord Clamping |
| 2019-05-05 | Before intervention | 250 | 159 | 63.6000000 | Delayed Cord Clamping |
| 2019-05-12 | Before intervention | 307 | 223 | 72.6384365 | Delayed Cord Clamping |
| 2019-05-19 | Before intervention | 149 | 90 | 60.4026846 | Delayed Cord Clamping |
| 2019-05-26 | Before intervention | 67 | 30 | 44.7761194 | Delayed Cord Clamping |
| 2019-06-02 | Before intervention | 194 | 155 | 79.8969072 | Delayed Cord Clamping |
| 2019-06-09 | Before intervention | 424 | 350 | 82.5471698 | Delayed Cord Clamping |
| 2019-06-16 | Before intervention | 513 | 395 | 76.9980507 | Delayed Cord Clamping |
| 2019-06-23 | Before intervention | 564 | 416 | 73.7588652 | Delayed Cord Clamping |
| 2019-06-30 | Before intervention | 661 | 458 | 69.2889561 | Delayed Cord Clamping |
| 2019-07-07 | Before intervention | 698 | 529 | 75.7879656 | Delayed Cord Clamping |
| 2019-07-14 | Before intervention | 715 | 528 | 73.8461538 | Delayed Cord Clamping |
| 2019-07-21 | Before intervention | 798 | 608 | 76.1904762 | Delayed Cord Clamping |
| 2019-07-28 | Before intervention | 866 | 622 | 71.8244804 | Delayed Cord Clamping |
| 2019-08-04 | Before intervention | 790 | 584 | 73.9240506 | Delayed Cord Clamping |
| 2019-08-11 | Before intervention | 675 | 535 | 79.2592593 | Delayed Cord Clamping |
| 2019-08-18 | Before intervention | 849 | 640 | 75.3828033 | Delayed Cord Clamping |
| 2019-08-25 | Before intervention | 852 | 700 | 82.1596244 | Delayed Cord Clamping |
| 2019-09-01 | Before intervention | 831 | 658 | 79.1817088 | Delayed Cord Clamping |
| 2019-09-08 | Before intervention | 798 | 648 | 81.2030075 | Delayed Cord Clamping |
| 2019-09-08 | After intervention | 15 | 13 | 86.6666667 | Delayed Cord Clamping |
| 2019-09-15 | Before intervention | 708 | 542 | 76.5536723 | Delayed Cord Clamping |
| 2019-09-15 | After intervention | 28 | 26 | 92.8571429 | Delayed Cord Clamping |
| 2019-09-22 | Before intervention | 675 | 530 | 78.5185185 | Delayed Cord Clamping |
| 2019-09-22 | After intervention | 36 | 31 | 86.1111111 | Delayed Cord Clamping |
| 2019-09-29 | Before intervention | 535 | 443 | 82.8037383 | Delayed Cord Clamping |
| 2019-09-29 | After intervention | 112 | 66 | 58.9285714 | Delayed Cord Clamping |
| 2019-10-06 | Before intervention | 492 | 423 | 85.9756098 | Delayed Cord Clamping |
| 2019-10-06 | After intervention | 106 | 63 | 59.4339623 | Delayed Cord Clamping |
| 2019-10-13 | Before intervention | 634 | 523 | 82.4921136 | Delayed Cord Clamping |
| 2019-10-13 | After intervention | 94 | 51 | 54.2553191 | Delayed Cord Clamping |
| 2019-10-20 | Before intervention | 650 | 528 | 81.2307692 | Delayed Cord Clamping |
| 2019-10-20 | After intervention | 70 | 27 | 38.5714286 | Delayed Cord Clamping |
| 2019-10-27 | Before intervention | 566 | 442 | 78.0918728 | Delayed Cord Clamping |
| 2019-10-27 | After intervention | 69 | 38 | 55.0724638 | Delayed Cord Clamping |
| 2019-11-03 | Before intervention | 628 | 520 | 82.8025478 | Delayed Cord Clamping |
| 2019-11-03 | After intervention | 85 | 61 | 71.7647059 | Delayed Cord Clamping |
| 2019-11-10 | Before intervention | 660 | 512 | 77.5757576 | Delayed Cord Clamping |
| 2019-11-10 | After intervention | 75 | 49 | 65.3333333 | Delayed Cord Clamping |
| 2019-11-17 | Before intervention | 619 | 476 | 76.8982229 | Delayed Cord Clamping |
| 2019-11-17 | After intervention | 77 | 53 | 68.8311688 | Delayed Cord Clamping |
| 2019-11-24 | Before intervention | 641 | 472 | 73.6349454 | Delayed Cord Clamping |
| 2019-11-24 | After intervention | 88 | 39 | 44.3181818 | Delayed Cord Clamping |
| 2019-12-01 | Before intervention | 678 | 500 | 73.7463127 | Delayed Cord Clamping |
| 2019-12-01 | After intervention | 44 | 32 | 72.7272727 | Delayed Cord Clamping |
| 2019-12-08 | Before intervention | 660 | 524 | 79.3939394 | Delayed Cord Clamping |
| 2019-12-08 | After intervention | 50 | 27 | 54.0000000 | Delayed Cord Clamping |
| 2019-12-15 | Before intervention | 644 | 480 | 74.5341615 | Delayed Cord Clamping |
| 2019-12-15 | After intervention | 57 | 30 | 52.6315789 | Delayed Cord Clamping |
| 2019-12-22 | Before intervention | 641 | 451 | 70.3588144 | Delayed Cord Clamping |
| 2019-12-22 | After intervention | 60 | 46 | 76.6666667 | Delayed Cord Clamping |
| 2019-12-29 | Before intervention | 656 | 397 | 60.5182927 | Delayed Cord Clamping |
| 2019-12-29 | After intervention | 44 | 33 | 75.0000000 | Delayed Cord Clamping |
| 2020-01-05 | Before intervention | 602 | 417 | 69.2691030 | Delayed Cord Clamping |
| 2020-01-05 | After intervention | 59 | 46 | 77.9661017 | Delayed Cord Clamping |
| 2020-01-12 | Before intervention | 577 | 366 | 63.4315425 | Delayed Cord Clamping |
| 2020-01-12 | After intervention | 69 | 48 | 69.5652174 | Delayed Cord Clamping |
| 2020-01-19 | Before intervention | 526 | 345 | 65.5893536 | Delayed Cord Clamping |
| 2020-01-19 | After intervention | 79 | 59 | 74.6835443 | Delayed Cord Clamping |
| 2020-01-26 | Before intervention | 413 | 263 | 63.6803874 | Delayed Cord Clamping |
| 2020-01-26 | After intervention | 202 | 128 | 63.3663366 | Delayed Cord Clamping |
| 2020-02-02 | Before intervention | 457 | 311 | 68.0525164 | Delayed Cord Clamping |
| 2020-02-02 | After intervention | 210 | 162 | 77.1428571 | Delayed Cord Clamping |
| 2020-02-09 | Before intervention | 417 | 286 | 68.5851319 | Delayed Cord Clamping |
| 2020-02-09 | After intervention | 195 | 150 | 76.9230769 | Delayed Cord Clamping |
| 2020-02-16 | Before intervention | 413 | 291 | 70.4600484 | Delayed Cord Clamping |
| 2020-02-16 | After intervention | 202 | 146 | 72.2772277 | Delayed Cord Clamping |
| 2020-02-23 | Before intervention | 403 | 321 | 79.6526055 | Delayed Cord Clamping |
| 2020-02-23 | After intervention | 208 | 191 | 91.8269231 | Delayed Cord Clamping |
| 2020-03-01 | Before intervention | 290 | 192 | 66.2068966 | Delayed Cord Clamping |
| 2020-03-01 | After intervention | 292 | 243 | 83.2191781 | Delayed Cord Clamping |
| 2020-03-08 | Before intervention | 234 | 152 | 64.9572650 | Delayed Cord Clamping |
| 2020-03-08 | After intervention | 308 | 267 | 86.6883117 | Delayed Cord Clamping |
| 2020-03-15 | Before intervention | 225 | 137 | 60.8888889 | Delayed Cord Clamping |
| 2020-03-15 | After intervention | 272 | 231 | 84.9264706 | Delayed Cord Clamping |
| 2020-03-22 | Before intervention | 118 | 81 | 68.6440678 | Delayed Cord Clamping |
| 2020-03-22 | After intervention | 100 | 90 | 90.0000000 | Delayed Cord Clamping |
| 2020-03-29 | Before intervention | 41 | 27 | 65.8536585 | Delayed Cord Clamping |
| 2020-03-29 | After intervention | 32 | 22 | 68.7500000 | Delayed Cord Clamping |
| 2020-04-05 | Before intervention | 72 | 43 | 59.7222222 | Delayed Cord Clamping |
| 2020-04-05 | After intervention | 72 | 50 | 69.4444444 | Delayed Cord Clamping |
| 2020-04-12 | Before intervention | 70 | 45 | 64.2857143 | Delayed Cord Clamping |
| 2020-04-12 | After intervention | 85 | 69 | 81.1764706 | Delayed Cord Clamping |
| 2020-04-19 | Before intervention | 76 | 41 | 53.9473684 | Delayed Cord Clamping |
| 2020-04-19 | After intervention | 110 | 88 | 80.0000000 | Delayed Cord Clamping |
| 2020-04-26 | Before intervention | 92 | 56 | 60.8695652 | Delayed Cord Clamping |
| 2020-04-26 | After intervention | 140 | 119 | 85.0000000 | Delayed Cord Clamping |
| 2020-05-03 | Before intervention | 70 | 51 | 72.8571429 | Delayed Cord Clamping |
| 2020-05-03 | After intervention | 143 | 119 | 83.2167832 | Delayed Cord Clamping |
| 2020-05-10 | Before intervention | 76 | 48 | 63.1578947 | Delayed Cord Clamping |
| 2020-05-10 | After intervention | 146 | 116 | 79.4520548 | Delayed Cord Clamping |
| 2020-05-17 | Before intervention | 75 | 39 | 52.0000000 | Delayed Cord Clamping |
| 2020-05-17 | After intervention | 130 | 98 | 75.3846154 | Delayed Cord Clamping |
| 2020-05-24 | Before intervention | 61 | 28 | 45.9016393 | Delayed Cord Clamping |
| 2020-05-24 | After intervention | 144 | 114 | 79.1666667 | Delayed Cord Clamping |
| 2020-05-31 | Before intervention | 96 | 64 | 66.6666667 | Delayed Cord Clamping |
| 2020-05-31 | After intervention | 126 | 104 | 82.5396825 | Delayed Cord Clamping |
| 2020-06-07 | Before intervention | 142 | 96 | 67.6056338 | Delayed Cord Clamping |
| 2020-06-07 | After intervention | 186 | 172 | 92.4731183 | Delayed Cord Clamping |
| 2020-06-14 | Before intervention | 166 | 92 | 55.4216867 | Delayed Cord Clamping |
| 2020-06-14 | After intervention | 235 | 210 | 89.3617021 | Delayed Cord Clamping |
| 2020-06-21 | Before intervention | 176 | 121 | 68.7500000 | Delayed Cord Clamping |
| 2020-06-21 | After intervention | 298 | 261 | 87.5838926 | Delayed Cord Clamping |
| 2020-06-28 | Before intervention | 121 | 43 | 35.5371901 | Delayed Cord Clamping |
| 2020-06-28 | After intervention | 448 | 393 | 87.7232143 | Delayed Cord Clamping |
| 2020-07-05 | Before intervention | 114 | 33 | 28.9473684 | Delayed Cord Clamping |
| 2020-07-05 | After intervention | 544 | 483 | 88.7867647 | Delayed Cord Clamping |
| 2020-07-12 | Before intervention | 106 | 34 | 32.0754717 | Delayed Cord Clamping |
| 2020-07-12 | After intervention | 535 | 487 | 91.0280374 | Delayed Cord Clamping |
| 2020-07-19 | Before intervention | 122 | 44 | 36.0655738 | Delayed Cord Clamping |
| 2020-07-19 | After intervention | 599 | 543 | 90.6510851 | Delayed Cord Clamping |
| 2020-07-26 | Before intervention | 96 | 52 | 54.1666667 | Delayed Cord Clamping |
| 2020-07-26 | After intervention | 608 | 546 | 89.8026316 | Delayed Cord Clamping |
| 2020-08-02 | Before intervention | 19 | 5 | 26.3157895 | Delayed Cord Clamping |
| 2020-08-02 | After intervention | 563 | 500 | 88.8099467 | Delayed Cord Clamping |
| 2020-08-09 | Before intervention | 65 | 17 | 26.1538462 | Delayed Cord Clamping |
| 2020-08-09 | After intervention | 505 | 460 | 91.0891089 | Delayed Cord Clamping |
| 2020-08-16 | Before intervention | 79 | 15 | 18.9873418 | Delayed Cord Clamping |
| 2020-08-16 | After intervention | 423 | 358 | 84.6335697 | Delayed Cord Clamping |
| 2020-08-23 | Before intervention | 101 | 20 | 19.8019802 | Delayed Cord Clamping |
| 2020-08-23 | After intervention | 467 | 403 | 86.2955032 | Delayed Cord Clamping |
| 2020-08-30 | Before intervention | 120 | 32 | 26.6666667 | Delayed Cord Clamping |
| 2020-08-30 | After intervention | 518 | 451 | 87.0656371 | Delayed Cord Clamping |
| 2020-09-06 | Before intervention | 118 | 45 | 38.1355932 | Delayed Cord Clamping |
| 2020-09-06 | After intervention | 470 | 396 | 84.2553191 | Delayed Cord Clamping |
| 2020-09-13 | Before intervention | 120 | 54 | 45.0000000 | Delayed Cord Clamping |
| 2020-09-13 | After intervention | 521 | 429 | 82.3416507 | Delayed Cord Clamping |
| 2020-09-20 | Before intervention | 133 | 54 | 40.6015038 | Delayed Cord Clamping |
| 2020-09-20 | After intervention | 591 | 510 | 86.2944162 | Delayed Cord Clamping |
| 2020-09-27 | Before intervention | 142 | 40 | 28.1690141 | Delayed Cord Clamping |
| 2020-09-27 | After intervention | 531 | 453 | 85.3107345 | Delayed Cord Clamping |
| 2020-10-04 | Before intervention | 113 | 43 | 38.0530973 | Delayed Cord Clamping |
| 2020-10-04 | After intervention | 570 | 450 | 78.9473684 | Delayed Cord Clamping |
| 2020-10-11 | After intervention | 677 | 522 | 77.1048744 | Delayed Cord Clamping |
| 2020-10-18 | After intervention | 739 | 544 | 73.6129905 | Delayed Cord Clamping |
| 2020-10-25 | After intervention | 714 | 529 | 74.0896359 | Delayed Cord Clamping |
| 2020-11-01 | After intervention | 670 | 462 | 68.9552239 | Delayed Cord Clamping |
| 2020-11-08 | After intervention | 711 | 489 | 68.7763713 | Delayed Cord Clamping |
| 2020-11-15 | After intervention | 758 | 544 | 71.7678100 | Delayed Cord Clamping |
| 2020-11-22 | After intervention | 828 | 600 | 72.4637681 | Delayed Cord Clamping |
| 2020-11-29 | After intervention | 801 | 570 | 71.1610487 | Delayed Cord Clamping |
| 2020-12-06 | After intervention | 743 | 523 | 70.3903096 | Delayed Cord Clamping |
| 2020-12-13 | After intervention | 757 | 516 | 68.1638045 | Delayed Cord Clamping |
| 2020-12-20 | After intervention | 763 | 570 | 74.7051114 | Delayed Cord Clamping |
| 2020-12-27 | After intervention | 730 | 527 | 72.1917808 | Delayed Cord Clamping |
| 2021-01-03 | After intervention | 752 | 543 | 72.2074468 | Delayed Cord Clamping |
| 2021-01-10 | After intervention | 732 | 536 | 73.2240437 | Delayed Cord Clamping |
| 2021-01-17 | After intervention | 676 | 508 | 75.1479290 | Delayed Cord Clamping |
| 2021-01-24 | After intervention | 581 | 432 | 74.3545611 | Delayed Cord Clamping |
| 2021-01-31 | After intervention | 623 | 478 | 76.7255217 | Delayed Cord Clamping |
| 2021-02-07 | After intervention | 619 | 442 | 71.4054927 | Delayed Cord Clamping |
| 2021-02-14 | After intervention | 557 | 389 | 69.8384201 | Delayed Cord Clamping |
| 2021-02-21 | After intervention | 572 | 418 | 73.0769231 | Delayed Cord Clamping |
| 2021-02-28 | After intervention | 508 | 366 | 72.0472441 | Delayed Cord Clamping |
| 2021-03-07 | After intervention | 499 | 386 | 77.3547094 | Delayed Cord Clamping |
| 2021-03-14 | After intervention | 462 | 327 | 70.7792208 | Delayed Cord Clamping |
| 2021-03-21 | After intervention | 503 | 381 | 75.7455268 | Delayed Cord Clamping |
| 2021-03-28 | After intervention | 261 | 193 | 73.9463602 | Delayed Cord Clamping |
| 2019-02-24 | Before intervention | 76 | 16 | 21.0526316 | Immediate Skin to Skin Contact |
| 2019-03-03 | Before intervention | 300 | 26 | 8.6666667 | Immediate Skin to Skin Contact |
| 2019-03-10 | Before intervention | 295 | 4 | 1.3559322 | Immediate Skin to Skin Contact |
| 2019-03-17 | Before intervention | 290 | 15 | 5.1724138 | Immediate Skin to Skin Contact |
| 2019-03-24 | Before intervention | 278 | 14 | 5.0359712 | Immediate Skin to Skin Contact |
| 2019-03-31 | Before intervention | 310 | 15 | 4.8387097 | Immediate Skin to Skin Contact |
| 2019-04-07 | Before intervention | 237 | 5 | 2.1097046 | Immediate Skin to Skin Contact |
| 2019-04-14 | Before intervention | 203 | 0 | 0.0000000 | Immediate Skin to Skin Contact |
| 2019-04-21 | Before intervention | 84 | 2 | 2.3809524 | Immediate Skin to Skin Contact |
| 2019-04-28 | Before intervention | 256 | 21 | 8.2031250 | Immediate Skin to Skin Contact |
| 2019-05-05 | Before intervention | 215 | 6 | 2.7906977 | Immediate Skin to Skin Contact |
| 2019-05-12 | Before intervention | 271 | 1 | 0.3690037 | Immediate Skin to Skin Contact |
| 2019-05-19 | Before intervention | 132 | 0 | 0.0000000 | Immediate Skin to Skin Contact |
| 2019-05-26 | Before intervention | 56 | 0 | 0.0000000 | Immediate Skin to Skin Contact |
| 2019-06-02 | Before intervention | 169 | 6 | 3.5502959 | Immediate Skin to Skin Contact |
| 2019-06-09 | Before intervention | 374 | 4 | 1.0695187 | Immediate Skin to Skin Contact |
| 2019-06-16 | Before intervention | 456 | 11 | 2.4122807 | Immediate Skin to Skin Contact |
| 2019-06-23 | Before intervention | 502 | 16 | 3.1872510 | Immediate Skin to Skin Contact |
| 2019-06-30 | Before intervention | 571 | 28 | 4.9036778 | Immediate Skin to Skin Contact |
| 2019-07-07 | Before intervention | 597 | 19 | 3.1825796 | Immediate Skin to Skin Contact |
| 2019-07-14 | Before intervention | 631 | 44 | 6.9730586 | Immediate Skin to Skin Contact |
| 2019-07-21 | Before intervention | 713 | 47 | 6.5918654 | Immediate Skin to Skin Contact |
| 2019-07-28 | Before intervention | 760 | 70 | 9.2105263 | Immediate Skin to Skin Contact |
| 2019-08-04 | Before intervention | 708 | 48 | 6.7796610 | Immediate Skin to Skin Contact |
| 2019-08-11 | Before intervention | 607 | 22 | 3.6243822 | Immediate Skin to Skin Contact |
| 2019-08-18 | Before intervention | 768 | 35 | 4.5572917 | Immediate Skin to Skin Contact |
| 2019-08-25 | Before intervention | 768 | 21 | 2.7343750 | Immediate Skin to Skin Contact |
| 2019-09-01 | Before intervention | 741 | 74 | 9.9865047 | Immediate Skin to Skin Contact |
| 2019-09-08 | Before intervention | 727 | 8 | 1.1004127 | Immediate Skin to Skin Contact |
| 2019-09-08 | After intervention | 12 | 10 | 83.3333333 | Immediate Skin to Skin Contact |
| 2019-09-15 | Before intervention | 629 | 4 | 0.6359300 | Immediate Skin to Skin Contact |
| 2019-09-15 | After intervention | 24 | 24 | 100.0000000 | Immediate Skin to Skin Contact |
| 2019-09-22 | Before intervention | 613 | 4 | 0.6525285 | Immediate Skin to Skin Contact |
| 2019-09-22 | After intervention | 32 | 20 | 62.5000000 | Immediate Skin to Skin Contact |
| 2019-09-29 | Before intervention | 474 | 9 | 1.8987342 | Immediate Skin to Skin Contact |
| 2019-09-29 | After intervention | 90 | 43 | 47.7777778 | Immediate Skin to Skin Contact |
| 2019-10-06 | Before intervention | 434 | 18 | 4.1474654 | Immediate Skin to Skin Contact |
| 2019-10-06 | After intervention | 98 | 22 | 22.4489796 | Immediate Skin to Skin Contact |
| 2019-10-13 | Before intervention | 573 | 12 | 2.0942408 | Immediate Skin to Skin Contact |
| 2019-10-13 | After intervention | 73 | 20 | 27.3972603 | Immediate Skin to Skin Contact |
| 2019-10-20 | Before intervention | 584 | 3 | 0.5136986 | Immediate Skin to Skin Contact |
| 2019-10-20 | After intervention | 59 | 13 | 22.0338983 | Immediate Skin to Skin Contact |
| 2019-10-27 | Before intervention | 500 | 8 | 1.6000000 | Immediate Skin to Skin Contact |
| 2019-10-27 | After intervention | 62 | 24 | 38.7096774 | Immediate Skin to Skin Contact |
| 2019-11-03 | Before intervention | 562 | 13 | 2.3131673 | Immediate Skin to Skin Contact |
| 2019-11-03 | After intervention | 68 | 32 | 47.0588235 | Immediate Skin to Skin Contact |
| 2019-11-10 | Before intervention | 593 | 3 | 0.5059022 | Immediate Skin to Skin Contact |
| 2019-11-10 | After intervention | 66 | 44 | 66.6666667 | Immediate Skin to Skin Contact |
| 2019-11-17 | Before intervention | 565 | 7 | 1.2389381 | Immediate Skin to Skin Contact |
| 2019-11-17 | After intervention | 64 | 40 | 62.5000000 | Immediate Skin to Skin Contact |
| 2019-11-24 | Before intervention | 594 | 8 | 1.3468013 | Immediate Skin to Skin Contact |
| 2019-11-24 | After intervention | 78 | 21 | 26.9230769 | Immediate Skin to Skin Contact |
| 2019-12-01 | Before intervention | 617 | 16 | 2.5931929 | Immediate Skin to Skin Contact |
| 2019-12-01 | After intervention | 38 | 23 | 60.5263158 | Immediate Skin to Skin Contact |
| 2019-12-08 | Before intervention | 599 | 15 | 2.5041736 | Immediate Skin to Skin Contact |
| 2019-12-08 | After intervention | 47 | 34 | 72.3404255 | Immediate Skin to Skin Contact |
| 2019-12-15 | Before intervention | 584 | 7 | 1.1986301 | Immediate Skin to Skin Contact |
| 2019-12-15 | After intervention | 45 | 31 | 68.8888889 | Immediate Skin to Skin Contact |
| 2019-12-22 | Before intervention | 577 | 5 | 0.8665511 | Immediate Skin to Skin Contact |
| 2019-12-22 | After intervention | 51 | 28 | 54.9019608 | Immediate Skin to Skin Contact |
| 2019-12-29 | Before intervention | 589 | 4 | 0.6791171 | Immediate Skin to Skin Contact |
| 2019-12-29 | After intervention | 36 | 24 | 66.6666667 | Immediate Skin to Skin Contact |
| 2020-01-05 | Before intervention | 535 | 7 | 1.3084112 | Immediate Skin to Skin Contact |
| 2020-01-05 | After intervention | 53 | 44 | 83.0188679 | Immediate Skin to Skin Contact |
| 2020-01-12 | Before intervention | 513 | 11 | 2.1442495 | Immediate Skin to Skin Contact |
| 2020-01-12 | After intervention | 60 | 42 | 70.0000000 | Immediate Skin to Skin Contact |
| 2020-01-19 | Before intervention | 465 | 19 | 4.0860215 | Immediate Skin to Skin Contact |
| 2020-01-19 | After intervention | 71 | 46 | 64.7887324 | Immediate Skin to Skin Contact |
| 2020-01-26 | Before intervention | 370 | 5 | 1.3513514 | Immediate Skin to Skin Contact |
| 2020-01-26 | After intervention | 190 | 40 | 21.0526316 | Immediate Skin to Skin Contact |
| 2020-02-02 | Before intervention | 398 | 17 | 4.2713568 | Immediate Skin to Skin Contact |
| 2020-02-02 | After intervention | 193 | 59 | 30.5699482 | Immediate Skin to Skin Contact |
| 2020-02-09 | Before intervention | 363 | 23 | 6.3360882 | Immediate Skin to Skin Contact |
| 2020-02-09 | After intervention | 186 | 30 | 16.1290323 | Immediate Skin to Skin Contact |
| 2020-02-16 | Before intervention | 355 | 25 | 7.0422535 | Immediate Skin to Skin Contact |
| 2020-02-16 | After intervention | 187 | 49 | 26.2032086 | Immediate Skin to Skin Contact |
| 2020-02-23 | Before intervention | 358 | 78 | 21.7877095 | Immediate Skin to Skin Contact |
| 2020-02-23 | After intervention | 199 | 87 | 43.7185930 | Immediate Skin to Skin Contact |
| 2020-03-01 | Before intervention | 254 | 33 | 12.9921260 | Immediate Skin to Skin Contact |
| 2020-03-01 | After intervention | 262 | 112 | 42.7480916 | Immediate Skin to Skin Contact |
| 2020-03-08 | Before intervention | 205 | 0 | 0.0000000 | Immediate Skin to Skin Contact |
| 2020-03-08 | After intervention | 295 | 182 | 61.6949153 | Immediate Skin to Skin Contact |
| 2020-03-15 | Before intervention | 194 | 0 | 0.0000000 | Immediate Skin to Skin Contact |
| 2020-03-15 | After intervention | 255 | 152 | 59.6078431 | Immediate Skin to Skin Contact |
| 2020-03-22 | Before intervention | 107 | 3 | 2.8037383 | Immediate Skin to Skin Contact |
| 2020-03-22 | After intervention | 96 | 71 | 73.9583333 | Immediate Skin to Skin Contact |
| 2020-03-29 | Before intervention | 37 | 1 | 2.7027027 | Immediate Skin to Skin Contact |
| 2020-03-29 | After intervention | 25 | 11 | 44.0000000 | Immediate Skin to Skin Contact |
| 2020-04-05 | Before intervention | 59 | 0 | 0.0000000 | Immediate Skin to Skin Contact |
| 2020-04-05 | After intervention | 63 | 45 | 71.4285714 | Immediate Skin to Skin Contact |
| 2020-04-12 | Before intervention | 59 | 0 | 0.0000000 | Immediate Skin to Skin Contact |
| 2020-04-12 | After intervention | 79 | 42 | 53.1645570 | Immediate Skin to Skin Contact |
| 2020-04-19 | Before intervention | 68 | 1 | 1.4705882 | Immediate Skin to Skin Contact |
| 2020-04-19 | After intervention | 105 | 68 | 64.7619048 | Immediate Skin to Skin Contact |
| 2020-04-26 | Before intervention | 79 | 0 | 0.0000000 | Immediate Skin to Skin Contact |
| 2020-04-26 | After intervention | 133 | 86 | 64.6616541 | Immediate Skin to Skin Contact |
| 2020-05-03 | Before intervention | 66 | 1 | 1.5151515 | Immediate Skin to Skin Contact |
| 2020-05-03 | After intervention | 139 | 95 | 68.3453237 | Immediate Skin to Skin Contact |
| 2020-05-10 | Before intervention | 68 | 2 | 2.9411765 | Immediate Skin to Skin Contact |
| 2020-05-10 | After intervention | 134 | 88 | 65.6716418 | Immediate Skin to Skin Contact |
| 2020-05-17 | Before intervention | 69 | 1 | 1.4492754 | Immediate Skin to Skin Contact |
| 2020-05-17 | After intervention | 119 | 63 | 52.9411765 | Immediate Skin to Skin Contact |
| 2020-05-24 | Before intervention | 57 | 0 | 0.0000000 | Immediate Skin to Skin Contact |
| 2020-05-24 | After intervention | 131 | 88 | 67.1755725 | Immediate Skin to Skin Contact |
| 2020-05-31 | Before intervention | 77 | 2 | 2.5974026 | Immediate Skin to Skin Contact |
| 2020-05-31 | After intervention | 114 | 65 | 57.0175439 | Immediate Skin to Skin Contact |
| 2020-06-07 | Before intervention | 124 | 6 | 4.8387097 | Immediate Skin to Skin Contact |
| 2020-06-07 | After intervention | 171 | 101 | 59.0643275 | Immediate Skin to Skin Contact |
| 2020-06-14 | Before intervention | 137 | 5 | 3.6496350 | Immediate Skin to Skin Contact |
| 2020-06-14 | After intervention | 221 | 147 | 66.5158371 | Immediate Skin to Skin Contact |
| 2020-06-21 | Before intervention | 157 | 32 | 20.3821656 | Immediate Skin to Skin Contact |
| 2020-06-21 | After intervention | 268 | 190 | 70.8955224 | Immediate Skin to Skin Contact |
| 2020-06-28 | Before intervention | 100 | 10 | 10.0000000 | Immediate Skin to Skin Contact |
| 2020-06-28 | After intervention | 396 | 284 | 71.7171717 | Immediate Skin to Skin Contact |
| 2020-07-05 | Before intervention | 88 | 7 | 7.9545455 | Immediate Skin to Skin Contact |
| 2020-07-05 | After intervention | 492 | 343 | 69.7154472 | Immediate Skin to Skin Contact |
| 2020-07-12 | Before intervention | 82 | 4 | 4.8780488 | Immediate Skin to Skin Contact |
| 2020-07-12 | After intervention | 488 | 354 | 72.5409836 | Immediate Skin to Skin Contact |
| 2020-07-19 | Before intervention | 101 | 1 | 0.9900990 | Immediate Skin to Skin Contact |
| 2020-07-19 | After intervention | 545 | 424 | 77.7981651 | Immediate Skin to Skin Contact |
| 2020-07-26 | Before intervention | 75 | 2 | 2.6666667 | Immediate Skin to Skin Contact |
| 2020-07-26 | After intervention | 554 | 435 | 78.5198556 | Immediate Skin to Skin Contact |
| 2020-08-02 | Before intervention | 14 | 1 | 7.1428571 | Immediate Skin to Skin Contact |
| 2020-08-02 | After intervention | 496 | 353 | 71.1693548 | Immediate Skin to Skin Contact |
| 2020-08-09 | Before intervention | 58 | 2 | 3.4482759 | Immediate Skin to Skin Contact |
| 2020-08-09 | After intervention | 452 | 328 | 72.5663717 | Immediate Skin to Skin Contact |
| 2020-08-16 | Before intervention | 64 | 0 | 0.0000000 | Immediate Skin to Skin Contact |
| 2020-08-16 | After intervention | 376 | 223 | 59.3085106 | Immediate Skin to Skin Contact |
| 2020-08-23 | Before intervention | 80 | 1 | 1.2500000 | Immediate Skin to Skin Contact |
| 2020-08-23 | After intervention | 414 | 277 | 66.9082126 | Immediate Skin to Skin Contact |
| 2020-08-30 | Before intervention | 96 | 2 | 2.0833333 | Immediate Skin to Skin Contact |
| 2020-08-30 | After intervention | 471 | 385 | 81.7409766 | Immediate Skin to Skin Contact |
| 2020-09-06 | Before intervention | 93 | 2 | 2.1505376 | Immediate Skin to Skin Contact |
| 2020-09-06 | After intervention | 430 | 331 | 76.9767442 | Immediate Skin to Skin Contact |
| 2020-09-13 | Before intervention | 94 | 0 | 0.0000000 | Immediate Skin to Skin Contact |
| 2020-09-13 | After intervention | 469 | 331 | 70.5756930 | Immediate Skin to Skin Contact |
| 2020-09-20 | Before intervention | 107 | 3 | 2.8037383 | Immediate Skin to Skin Contact |
| 2020-09-20 | After intervention | 537 | 343 | 63.8733706 | Immediate Skin to Skin Contact |
| 2020-09-27 | Before intervention | 113 | 0 | 0.0000000 | Immediate Skin to Skin Contact |
| 2020-09-27 | After intervention | 479 | 300 | 62.6304802 | Immediate Skin to Skin Contact |
| 2020-10-04 | Before intervention | 87 | 24 | 27.5862069 | Immediate Skin to Skin Contact |
| 2020-10-04 | After intervention | 517 | 330 | 63.8297872 | Immediate Skin to Skin Contact |
| 2020-10-11 | After intervention | 623 | 344 | 55.2166934 | Immediate Skin to Skin Contact |
| 2020-10-18 | After intervention | 678 | 363 | 53.5398230 | Immediate Skin to Skin Contact |
| 2020-10-25 | After intervention | 663 | 314 | 47.3604827 | Immediate Skin to Skin Contact |
| 2020-11-01 | After intervention | 610 | 323 | 52.9508197 | Immediate Skin to Skin Contact |
| 2020-11-08 | After intervention | 625 | 349 | 55.8400000 | Immediate Skin to Skin Contact |
| 2020-11-15 | After intervention | 699 | 399 | 57.0815451 | Immediate Skin to Skin Contact |
| 2020-11-22 | After intervention | 744 | 380 | 51.0752688 | Immediate Skin to Skin Contact |
| 2020-11-29 | After intervention | 739 | 403 | 54.5331529 | Immediate Skin to Skin Contact |
| 2020-12-06 | After intervention | 673 | 375 | 55.7206538 | Immediate Skin to Skin Contact |
| 2020-12-13 | After intervention | 686 | 370 | 53.9358601 | Immediate Skin to Skin Contact |
| 2020-12-20 | After intervention | 685 | 380 | 55.4744526 | Immediate Skin to Skin Contact |
| 2020-12-27 | After intervention | 644 | 378 | 58.6956522 | Immediate Skin to Skin Contact |
| 2021-01-03 | After intervention | 673 | 410 | 60.9212481 | Immediate Skin to Skin Contact |
| 2021-01-10 | After intervention | 650 | 346 | 53.2307692 | Immediate Skin to Skin Contact |
| 2021-01-17 | After intervention | 617 | 361 | 58.5089141 | Immediate Skin to Skin Contact |
| 2021-01-24 | After intervention | 537 | 277 | 51.5828678 | Immediate Skin to Skin Contact |
| 2021-01-31 | After intervention | 560 | 305 | 54.4642857 | Immediate Skin to Skin Contact |
| 2021-02-07 | After intervention | 551 | 317 | 57.5317604 | Immediate Skin to Skin Contact |
| 2021-02-14 | After intervention | 497 | 301 | 60.5633803 | Immediate Skin to Skin Contact |
| 2021-02-21 | After intervention | 511 | 324 | 63.4050881 | Immediate Skin to Skin Contact |
| 2021-02-28 | After intervention | 454 | 297 | 65.4185022 | Immediate Skin to Skin Contact |
| 2021-03-07 | After intervention | 453 | 351 | 77.4834437 | Immediate Skin to Skin Contact |
| 2021-03-14 | After intervention | 405 | 289 | 71.3580247 | Immediate Skin to Skin Contact |
| 2021-03-21 | After intervention | 452 | 339 | 75.0000000 | Immediate Skin to Skin Contact |
| 2021-03-28 | After intervention | 235 | 131 | 55.7446809 | Immediate Skin to Skin Contact |
| 2019-02-24 | Before intervention | 86 | 1 | 1.1627907 | Early Breast Feeding |
| 2019-03-03 | Before intervention | 325 | 14 | 4.3076923 | Early Breast Feeding |
| 2019-03-10 | Before intervention | 322 | 14 | 4.3478261 | Early Breast Feeding |
| 2019-03-17 | Before intervention | 317 | 12 | 3.7854890 | Early Breast Feeding |
| 2019-03-24 | Before intervention | 311 | 11 | 3.5369775 | Early Breast Feeding |
| 2019-03-31 | Before intervention | 350 | 26 | 7.4285714 | Early Breast Feeding |
| 2019-04-07 | Before intervention | 270 | 13 | 4.8148148 | Early Breast Feeding |
| 2019-04-14 | Before intervention | 220 | 17 | 7.7272727 | Early Breast Feeding |
| 2019-04-21 | Before intervention | 91 | 10 | 10.9890110 | Early Breast Feeding |
| 2019-04-28 | Before intervention | 299 | 10 | 3.3444816 | Early Breast Feeding |
| 2019-05-05 | Before intervention | 250 | 14 | 5.6000000 | Early Breast Feeding |
| 2019-05-12 | Before intervention | 307 | 25 | 8.1433225 | Early Breast Feeding |
| 2019-05-19 | Before intervention | 149 | 17 | 11.4093960 | Early Breast Feeding |
| 2019-05-26 | Before intervention | 67 | 1 | 1.4925373 | Early Breast Feeding |
| 2019-06-02 | Before intervention | 194 | 21 | 10.8247423 | Early Breast Feeding |
| 2019-06-09 | Before intervention | 424 | 23 | 5.4245283 | Early Breast Feeding |
| 2019-06-16 | Before intervention | 513 | 19 | 3.7037037 | Early Breast Feeding |
| 2019-06-23 | Before intervention | 564 | 36 | 6.3829787 | Early Breast Feeding |
| 2019-06-30 | Before intervention | 661 | 68 | 10.2874433 | Early Breast Feeding |
| 2019-07-07 | Before intervention | 698 | 52 | 7.4498567 | Early Breast Feeding |
| 2019-07-14 | Before intervention | 715 | 32 | 4.4755245 | Early Breast Feeding |
| 2019-07-21 | Before intervention | 798 | 27 | 3.3834586 | Early Breast Feeding |
| 2019-07-28 | Before intervention | 866 | 54 | 6.2355658 | Early Breast Feeding |
| 2019-08-04 | Before intervention | 790 | 45 | 5.6962025 | Early Breast Feeding |
| 2019-08-11 | Before intervention | 675 | 14 | 2.0740741 | Early Breast Feeding |
| 2019-08-18 | Before intervention | 849 | 21 | 2.4734982 | Early Breast Feeding |
| 2019-08-25 | Before intervention | 852 | 20 | 2.3474178 | Early Breast Feeding |
| 2019-09-01 | Before intervention | 831 | 38 | 4.5728039 | Early Breast Feeding |
| 2019-09-08 | Before intervention | 798 | 21 | 2.6315789 | Early Breast Feeding |
| 2019-09-08 | After intervention | 15 | 7 | 46.6666667 | Early Breast Feeding |
| 2019-09-15 | Before intervention | 708 | 23 | 3.2485876 | Early Breast Feeding |
| 2019-09-15 | After intervention | 28 | 7 | 25.0000000 | Early Breast Feeding |
| 2019-09-22 | Before intervention | 675 | 10 | 1.4814815 | Early Breast Feeding |
| 2019-09-22 | After intervention | 36 | 9 | 25.0000000 | Early Breast Feeding |
| 2019-09-29 | Before intervention | 535 | 24 | 4.4859813 | Early Breast Feeding |
| 2019-09-29 | After intervention | 112 | 15 | 13.3928571 | Early Breast Feeding |
| 2019-10-06 | Before intervention | 492 | 25 | 5.0813008 | Early Breast Feeding |
| 2019-10-06 | After intervention | 106 | 18 | 16.9811321 | Early Breast Feeding |
| 2019-10-13 | Before intervention | 634 | 42 | 6.6246057 | Early Breast Feeding |
| 2019-10-13 | After intervention | 94 | 15 | 15.9574468 | Early Breast Feeding |
| 2019-10-20 | Before intervention | 650 | 29 | 4.4615385 | Early Breast Feeding |
| 2019-10-20 | After intervention | 70 | 13 | 18.5714286 | Early Breast Feeding |
| 2019-10-27 | Before intervention | 566 | 19 | 3.3568905 | Early Breast Feeding |
| 2019-10-27 | After intervention | 69 | 20 | 28.9855072 | Early Breast Feeding |
| 2019-11-03 | Before intervention | 628 | 38 | 6.0509554 | Early Breast Feeding |
| 2019-11-03 | After intervention | 85 | 34 | 40.0000000 | Early Breast Feeding |
| 2019-11-10 | Before intervention | 660 | 28 | 4.2424242 | Early Breast Feeding |
| 2019-11-10 | After intervention | 75 | 31 | 41.3333333 | Early Breast Feeding |
| 2019-11-17 | Before intervention | 619 | 45 | 7.2697900 | Early Breast Feeding |
| 2019-11-17 | After intervention | 77 | 28 | 36.3636364 | Early Breast Feeding |
| 2019-11-24 | Before intervention | 641 | 32 | 4.9921997 | Early Breast Feeding |
| 2019-11-24 | After intervention | 88 | 14 | 15.9090909 | Early Breast Feeding |
| 2019-12-01 | Before intervention | 678 | 96 | 14.1592920 | Early Breast Feeding |
| 2019-12-01 | After intervention | 44 | 10 | 22.7272727 | Early Breast Feeding |
| 2019-12-08 | Before intervention | 660 | 167 | 25.3030303 | Early Breast Feeding |
| 2019-12-08 | After intervention | 50 | 21 | 42.0000000 | Early Breast Feeding |
| 2019-12-15 | Before intervention | 644 | 150 | 23.2919255 | Early Breast Feeding |
| 2019-12-15 | After intervention | 57 | 24 | 42.1052632 | Early Breast Feeding |
| 2019-12-22 | Before intervention | 641 | 152 | 23.7129485 | Early Breast Feeding |
| 2019-12-22 | After intervention | 60 | 33 | 55.0000000 | Early Breast Feeding |
| 2019-12-29 | Before intervention | 656 | 139 | 21.1890244 | Early Breast Feeding |
| 2019-12-29 | After intervention | 44 | 19 | 43.1818182 | Early Breast Feeding |
| 2020-01-05 | Before intervention | 602 | 141 | 23.4219269 | Early Breast Feeding |
| 2020-01-05 | After intervention | 59 | 38 | 64.4067797 | Early Breast Feeding |
| 2020-01-12 | Before intervention | 577 | 138 | 23.9168111 | Early Breast Feeding |
| 2020-01-12 | After intervention | 69 | 44 | 63.7681159 | Early Breast Feeding |
| 2020-01-19 | Before intervention | 526 | 168 | 31.9391635 | Early Breast Feeding |
| 2020-01-19 | After intervention | 79 | 53 | 67.0886076 | Early Breast Feeding |
| 2020-01-26 | Before intervention | 413 | 176 | 42.6150121 | Early Breast Feeding |
| 2020-01-26 | After intervention | 202 | 96 | 47.5247525 | Early Breast Feeding |
| 2020-02-02 | Before intervention | 457 | 165 | 36.1050328 | Early Breast Feeding |
| 2020-02-02 | After intervention | 210 | 119 | 56.6666667 | Early Breast Feeding |
| 2020-02-09 | Before intervention | 417 | 157 | 37.6498801 | Early Breast Feeding |
| 2020-02-09 | After intervention | 195 | 105 | 53.8461538 | Early Breast Feeding |
| 2020-02-16 | Before intervention | 413 | 165 | 39.9515738 | Early Breast Feeding |
| 2020-02-16 | After intervention | 202 | 108 | 53.4653465 | Early Breast Feeding |
| 2020-02-23 | Before intervention | 403 | 176 | 43.6724566 | Early Breast Feeding |
| 2020-02-23 | After intervention | 208 | 156 | 75.0000000 | Early Breast Feeding |
| 2020-03-01 | Before intervention | 290 | 131 | 45.1724138 | Early Breast Feeding |
| 2020-03-01 | After intervention | 292 | 173 | 59.2465753 | Early Breast Feeding |
| 2020-03-08 | Before intervention | 234 | 75 | 32.0512821 | Early Breast Feeding |
| 2020-03-08 | After intervention | 308 | 189 | 61.3636364 | Early Breast Feeding |
| 2020-03-15 | Before intervention | 225 | 76 | 33.7777778 | Early Breast Feeding |
| 2020-03-15 | After intervention | 272 | 182 | 66.9117647 | Early Breast Feeding |
| 2020-03-22 | Before intervention | 118 | 26 | 22.0338983 | Early Breast Feeding |
| 2020-03-22 | After intervention | 100 | 74 | 74.0000000 | Early Breast Feeding |
| 2020-03-29 | Before intervention | 41 | 11 | 26.8292683 | Early Breast Feeding |
| 2020-03-29 | After intervention | 32 | 17 | 53.1250000 | Early Breast Feeding |
| 2020-04-05 | Before intervention | 72 | 15 | 20.8333333 | Early Breast Feeding |
| 2020-04-05 | After intervention | 72 | 43 | 59.7222222 | Early Breast Feeding |
| 2020-04-12 | Before intervention | 70 | 18 | 25.7142857 | Early Breast Feeding |
| 2020-04-12 | After intervention | 85 | 42 | 49.4117647 | Early Breast Feeding |
| 2020-04-19 | Before intervention | 76 | 17 | 22.3684211 | Early Breast Feeding |
| 2020-04-19 | After intervention | 110 | 73 | 66.3636364 | Early Breast Feeding |
| 2020-04-26 | Before intervention | 92 | 19 | 20.6521739 | Early Breast Feeding |
| 2020-04-26 | After intervention | 140 | 73 | 52.1428571 | Early Breast Feeding |
| 2020-05-03 | Before intervention | 70 | 7 | 10.0000000 | Early Breast Feeding |
| 2020-05-03 | After intervention | 143 | 85 | 59.4405594 | Early Breast Feeding |
| 2020-05-10 | Before intervention | 76 | 50 | 65.7894737 | Early Breast Feeding |
| 2020-05-10 | After intervention | 146 | 85 | 58.2191781 | Early Breast Feeding |
| 2020-05-17 | Before intervention | 75 | 39 | 52.0000000 | Early Breast Feeding |
| 2020-05-17 | After intervention | 130 | 80 | 61.5384615 | Early Breast Feeding |
| 2020-05-24 | Before intervention | 61 | 36 | 59.0163934 | Early Breast Feeding |
| 2020-05-24 | After intervention | 144 | 99 | 68.7500000 | Early Breast Feeding |
| 2020-05-31 | Before intervention | 96 | 41 | 42.7083333 | Early Breast Feeding |
| 2020-05-31 | After intervention | 126 | 80 | 63.4920635 | Early Breast Feeding |
| 2020-06-07 | Before intervention | 142 | 69 | 48.5915493 | Early Breast Feeding |
| 2020-06-07 | After intervention | 186 | 110 | 59.1397849 | Early Breast Feeding |
| 2020-06-14 | Before intervention | 166 | 70 | 42.1686747 | Early Breast Feeding |
| 2020-06-14 | After intervention | 235 | 139 | 59.1489362 | Early Breast Feeding |
| 2020-06-21 | Before intervention | 176 | 88 | 50.0000000 | Early Breast Feeding |
| 2020-06-21 | After intervention | 298 | 187 | 62.7516779 | Early Breast Feeding |
| 2020-06-28 | Before intervention | 121 | 45 | 37.1900826 | Early Breast Feeding |
| 2020-06-28 | After intervention | 448 | 242 | 54.0178571 | Early Breast Feeding |
| 2020-07-05 | Before intervention | 114 | 36 | 31.5789474 | Early Breast Feeding |
| 2020-07-05 | After intervention | 544 | 282 | 51.8382353 | Early Breast Feeding |
| 2020-07-12 | Before intervention | 106 | 44 | 41.5094340 | Early Breast Feeding |
| 2020-07-12 | After intervention | 535 | 306 | 57.1962617 | Early Breast Feeding |
| 2020-07-19 | Before intervention | 122 | 42 | 34.4262295 | Early Breast Feeding |
| 2020-07-19 | After intervention | 599 | 330 | 55.0918197 | Early Breast Feeding |
| 2020-07-26 | Before intervention | 96 | 33 | 34.3750000 | Early Breast Feeding |
| 2020-07-26 | After intervention | 608 | 331 | 54.4407895 | Early Breast Feeding |
| 2020-08-02 | Before intervention | 19 | 11 | 57.8947368 | Early Breast Feeding |
| 2020-08-02 | After intervention | 563 | 324 | 57.5488455 | Early Breast Feeding |
| 2020-08-09 | Before intervention | 65 | 10 | 15.3846154 | Early Breast Feeding |
| 2020-08-09 | After intervention | 505 | 237 | 46.9306931 | Early Breast Feeding |
| 2020-08-16 | Before intervention | 79 | 22 | 27.8481013 | Early Breast Feeding |
| 2020-08-16 | After intervention | 423 | 163 | 38.5342790 | Early Breast Feeding |
| 2020-08-23 | Before intervention | 101 | 20 | 19.8019802 | Early Breast Feeding |
| 2020-08-23 | After intervention | 467 | 250 | 53.5331906 | Early Breast Feeding |
| 2020-08-30 | Before intervention | 120 | 42 | 35.0000000 | Early Breast Feeding |
| 2020-08-30 | After intervention | 518 | 289 | 55.7915058 | Early Breast Feeding |
| 2020-09-06 | Before intervention | 118 | 46 | 38.9830508 | Early Breast Feeding |
| 2020-09-06 | After intervention | 470 | 265 | 56.3829787 | Early Breast Feeding |
| 2020-09-13 | Before intervention | 120 | 42 | 35.0000000 | Early Breast Feeding |
| 2020-09-13 | After intervention | 521 | 244 | 46.8330134 | Early Breast Feeding |
| 2020-09-20 | Before intervention | 133 | 71 | 53.3834586 | Early Breast Feeding |
| 2020-09-20 | After intervention | 591 | 344 | 58.2064298 | Early Breast Feeding |
| 2020-09-27 | Before intervention | 142 | 81 | 57.0422535 | Early Breast Feeding |
| 2020-09-27 | After intervention | 531 | 284 | 53.4839925 | Early Breast Feeding |
| 2020-10-04 | Before intervention | 113 | 63 | 55.7522124 | Early Breast Feeding |
| 2020-10-04 | After intervention | 570 | 298 | 52.2807018 | Early Breast Feeding |
| 2020-10-11 | After intervention | 677 | 357 | 52.7326440 | Early Breast Feeding |
| 2020-10-18 | After intervention | 739 | 447 | 60.4871448 | Early Breast Feeding |
| 2020-10-25 | After intervention | 714 | 434 | 60.7843137 | Early Breast Feeding |
| 2020-11-01 | After intervention | 670 | 384 | 57.3134328 | Early Breast Feeding |
| 2020-11-08 | After intervention | 711 | 402 | 56.5400844 | Early Breast Feeding |
| 2020-11-15 | After intervention | 758 | 452 | 59.6306069 | Early Breast Feeding |
| 2020-11-22 | After intervention | 828 | 483 | 58.3333333 | Early Breast Feeding |
| 2020-11-29 | After intervention | 801 | 485 | 60.5493134 | Early Breast Feeding |
| 2020-12-06 | After intervention | 743 | 418 | 56.2584118 | Early Breast Feeding |
| 2020-12-13 | After intervention | 757 | 412 | 54.4253633 | Early Breast Feeding |
| 2020-12-20 | After intervention | 763 | 442 | 57.9292267 | Early Breast Feeding |
| 2020-12-27 | After intervention | 730 | 440 | 60.2739726 | Early Breast Feeding |
| 2021-01-03 | After intervention | 752 | 459 | 61.0372340 | Early Breast Feeding |
| 2021-01-10 | After intervention | 732 | 435 | 59.4262295 | Early Breast Feeding |
| 2021-01-17 | After intervention | 676 | 405 | 59.9112426 | Early Breast Feeding |
| 2021-01-24 | After intervention | 581 | 343 | 59.0361446 | Early Breast Feeding |
| 2021-01-31 | After intervention | 623 | 372 | 59.7110754 | Early Breast Feeding |
| 2021-02-07 | After intervention | 619 | 407 | 65.7512116 | Early Breast Feeding |
| 2021-02-14 | After intervention | 557 | 372 | 66.7863555 | Early Breast Feeding |
| 2021-02-21 | After intervention | 572 | 411 | 71.8531469 | Early Breast Feeding |
| 2021-02-28 | After intervention | 508 | 359 | 70.6692913 | Early Breast Feeding |
| 2021-03-07 | After intervention | 499 | 402 | 80.5611222 | Early Breast Feeding |
| 2021-03-14 | After intervention | 462 | 352 | 76.1904762 | Early Breast Feeding |
| 2021-03-21 | After intervention | 503 | 369 | 73.3598410 | Early Breast Feeding |
| 2021-03-28 | After intervention | 261 | 191 | 73.1800766 | Early Breast Feeding |
