# Supplementary material for: Effect of meso-and micro-level quality improvement interventions to improve intrapartum care and birth outcome in Nepal- stepped wedge cluster randomized trial
Source: AJOG Glob Rep. 2026 Sep 16;6(4):100697. doi: 10.1016/j.xagr.2026.100697 (PMC13677397; doi:10.1016/j.xagr.2026.100697)
Supplement: Supplementary file 3 [file mmc1.docx]

Supplementary Figure 1: Stepped-wedge Implementation of the SUSTAIN package
